# Integrated management strategies for benign prostatic hyperplasia

**DOI:** 10.3389/fruro.2025.1641171

**Published:** 2025-10-13

**Authors:** Yingchen Zhou, Qiao Luo, Ruixiao Wang, Dongxiang Zheng, Yaqing Xiong, Jiao Zuo, Shusheng Wang, Liang Zhong

**Affiliations:** ^1^ Department of Urology Surgery, Zhongshan Hospital of Traditional Chinese Medicine, Zhongshan, China; ^2^ School of Pharmacy, Faculty of Medicine, Macau University of Science and Technology, Macau, Macao SAR, China; ^3^ The Synthesis Team of the Transformation Research Institute, Shenzhen Second People’s Hospital, Shenzhen, China; ^4^ Department of Urology Surgery, Guangdong Provincial Hospital of Traditional Chinese Medicine, Guangzhou, China

**Keywords:** benign prostatic hyperplasia, medications, minimally invasive surgical therapies, lower urinary tract symptoms, prostate

## Abstract

Benign prostatic hyperplasia (BPH) is a common condition in aging men, leading to lower urinary tract symptoms (LUTS) that affect quality of life. Treatment options have evolved from invasive surgeries to a combination of pharmacological therapies, minimally invasive surgical therapies (MISTs), and standard surgical procedures. Medications such as α-blockers, 5-alpha reductase inhibitors (5-ARIs), and phosphodiesterase-5 inhibitors (PDE5i) are the first-line treatment for mild-to-moderate BPH, while MISTs like Rezūm, UroLift, Aquablation, and prostatic artery embolization (PAE) provide less invasive alternatives with shorter recovery times. For larger prostates, TURP and HoLEP remain the gold standards, offering effective long-term symptom relief despite some risks. Future advancements in BPH treatment focus on robotic-assisted surgery, AI-guided treatment selection, hybrid therapies, and regenerative medicine, aiming to enhance precision, reduce complications, and improve patient outcomes. This review summarizes current BPH management strategies and explores future innovations in the field.

## 1 Introduction

BPH is a prevalent condition among older men, affecting more than half of those over the age of 50. Its frequency rises with age, becoming more common as men grow older (1). Studies show that approximately 50% of men over 60 years of age experience varying degrees of bladder outlet obstruction, leading to LUTS, which significantly affect their quality of life (QoL) (2). According to the European Association of Urology (EAU) guidelines, the treatment of LUTS should follow a stepwise approach. Initially, conservative management, including behavioral and dietary changes, is recommended, as approximately 79% of LUTS patients remain clinically stable over a period of five years (3). If symptoms do not improve effectively, drug treatment can be considered, tailored to prostate volume and symptom severity, which may involve single or combination therapies. For patients with urinary retention, recurrent urinary tract infections, bladder stones, recurrent massive hematuria, renal insufficiency, or overflow incontinence, surgical intervention is generally considered an effective option. However, both medical and surgical treatments may come with side effects, particularly affecting ejaculation and sexual function. Therefore, treatment choices must carefully consider the patient’s symptoms, condition, and associated risks. With the growing aging population, patients with multiple comorbidities are often not suitable candidates for surgical treatment, highlighting the need for alternative approaches. In this context, prostatic stents play a crucial role by offering a minimally invasive treatment option that can be performed in an outpatient setting, possibly under local anesthesia. Since the 1980s, prostatic stents have been widely used in clinical practice. These stents are placed temporarily or permanently in the prostatic urethra to compress the prostate tissue and relieve bladder outlet obstruction (BOO). The insertion of stents can be done on an outpatient basis using regional or local anesthesia, providing rapid symptom relief, but they require a functional detrusor muscle. Prostatic stents come in various materials and shapes and are categorized as either permanent (epithelializing) or temporary (non-epithelializing). Some materials inhibit epithelial growth, making removal easier. Temporary stents may be either biostable or biodegradable, while permanent stents are biocompatible, promoting epithelialization.

Given these considerations, this article aims to provide a comprehensive overview of BPH management with a particular focus on the role of prostatic stents. We will discuss their mechanism of action, clinical indications, advantages, limitations, and potential future developments, thereby highlighting their value as a minimally invasive alternative in the treatment paradigm for BPH—especially among elderly patients with significant comorbidities.

### 1.1 Pathology of BPH

BPH is a common pathological condition in elderly men, primarily characterized by the hyperplasia of prostate glands and stroma. Its pathological mechanisms are not yet fully understood, but studies have shown that the occurrence of BPH is closely related to several factors, including the action of androgens, chronic inflammatory responses, infiltration of immune cells, metabolic disorders, and epigenetic changes. These factors not only act independently but also interact in complex ways to promote the development and progression of BPH. The following sections discuss these major mechanisms in detail.

#### 1.1.1 The role of androgens

Androgens, especially dihydrotestosterone (DHT), play a crucial role in the development of BPH (4). DHT is derived from testosterone through the action of 5-alpha reductase and is the primary androgen in the prostate. Although testosterone levels decline with age in elderly men, DHT levels in the prostate remain relatively high even under low testosterone conditions, which is sufficient to promote the hyperplastic process of the prostate (5). DHT binds to androgen receptors (AR) in prostate cells, activating a series of signaling pathways that regulate cell proliferation, matrix remodeling, and apoptosis (6). Recent studies have shown that DHT not only promotes epithelial cell proliferation through direct activation of AR but also regulates the proliferation of stromal cells and fibrosis, further driving the development of BPH (7). Moreover, the action of DHT is not a singular AR activation process. Numerous studies have indicated that DHT influences prostate hyperplasia through interactions with other hormones, such as estrogen. Estrogen may indirectly participate in the development of BPH by modulating androgen effects. These complex interactions between hormones form a multi-dimensional regulatory network of prostate hyperplasia, further highlighting the complexity of the pathological mechanisms of BPH (8).

#### 1.1.2 Prostatic tissue hyperplastic response

In the pathological process of BPH, tissue hyperplasia of the prostate is the most prominent feature, particularly in the region surrounding the urethra. The process of prostatic hyperplasia involves not only the proliferation of epithelial cells but also the proliferation and fibrosis of stromal cells. Epithelial cell proliferation leads to glandular expansion, forming multiple acini, which increases the prostate volume (9). On the other hand, the proliferation of stromal cells results in the remodeling of the extracellular matrix, and the excessive deposition of collagen worsens prostate sclerosis, affecting its elasticity and leading to increased intra-urethral pressure (10). The elevated intra-urethral pressure causes symptoms such as difficulty urinating and increased urinary flow resistance. Additionally, remodeling of the extracellular matrix continues not only during the early stages of hyperplasia but also throughout the chronic phase of BPH. The proliferating stromal cells secrete growth factors such as transforming growth factor-beta (TGF-β) and epidermal growth factor (EGF), which further accelerate the fibrosis process. TGF-β plays a particularly prominent role in fibrosis, as it induces fibroblast proliferation and promotes collagen synthesis, thereby accelerating structural changes in the prostate (11, 12).

#### 1.1.3 Chronic inflammatory response

In the prostate tissue of BPH, immune cells, especially macrophages and T cells, are the main mediators of inflammation. Macrophages secrete various pro-inflammatory factors, such as tumor necrosis factor-α (TNF-α) and interleukin-1 (IL - 1), which not only intensify the local inflammatory response but also activate the androgen receptor (AR) signaling pathway in prostate cells, promoting cell proliferation and local tissue remodeling (13, 14). At the same time, T cells, particularly T helper cell (Th1 and Th2) subsets, release cytokines that significantly affect prostate hyperplasia. Th1 cells primarily secrete INF-γ and IL - 2, which have strong pro-inflammatory effects, while as BPH progresses, Th2 cells gradually dominate and secrete IL - 4 and IL - 13, which promote fibrosis in the prostate (15, 16). These cytokines act together, worsening the local inflammatory environment and exacerbating prostate hyperplasia. In addition to cytokines, the inflammatory response also activates oxidative stress, increasing the levels of reactive oxygen species (ROS) in prostate tissue, further exacerbating prostate hyperplasia. The generation of ROS is closely related to the hypoxic environment in BPH, as the low oxygen state promotes oxidative stress. ROS can promote cell damage, cell proliferation, and matrix remodeling through multiple mechanisms. Studies have shown that the hypoxic environment not only induces ROS generation to activate the AR signaling pathway but also further enhances the persistence of the inflammatory response (17, 18). ROS interacts with TGF-β, further driving the fibrosis process in the prostate, forming a vicious cycle. TGF-β plays a central role in this process by promoting fibroblast proliferation and collagen synthesis, accelerating the fibrosis of the prostate matrix, increasing tissue stiffness, and raising intra-urethral pressure, thereby worsening the symptoms of BPH (19–21). Chronic inflammation not only aggravates damage to prostate tissue but also provides a more favorable microenvironment for prostate hyperplasia. Under the influence of chronic inflammation, immune cell infiltration, cytokine secretion, and oxidative stress act together in the prostate tissue, constructing a vicious cycle (22). These factors enhance cell proliferation, promote matrix deposition, and fibrosis, ultimately driving the progression of BPH. Furthermore, as prostate hyperplasia progresses, changes in the local tissue’s hemodynamics and inadequate oxygen supply aggravate the hypoxic state, further promoting inflammation and ROS generation, thus accelerating the hyperplastic process in the prostate (21).

#### 1.1.4 Metabolic disorders

Metabolic disorders, particularly obesity and diabetes, have been strongly linked to the occurrence and progression of BPH (23, 24). Obesity plays a central role in exacerbating BPH by increasing the levels of fatty acids in the body, which in turn activate pro-inflammatory cytokines and oxidative stress pathways (25). These processes contribute significantly to the acceleration of prostate hyperplasia. Specifically, obesity and elevated insulin levels trigger metabolic inflammation that alters the metabolic environment of the prostate, enhancing the proliferative response of prostate tissues (26). This metabolic dysregulation not only stimulates the growth of prostate epithelial cells but also promotes stromal remodeling and fibrosis, making the prostate more prone to hyperplasia. Moreover, the alteration of adipocyte factor secretion due to obesity further supports the fibrotic processes and cell proliferation within the prostate. Increased secretion of adipokines such as leptin and resistin can contribute to the inflammatory state in the prostate, thereby facilitating the fibrotic changes and increasing the likelihood of BPH development. Systemic chronic inflammation, driven by these metabolic disturbances, creates a more favorable environment for BPH progression. As the disease advances, the interplay between metabolic dysregulation and local inflammatory responses accelerates the chronic nature of BPH, thereby further enhancing both epithelial and stromal proliferation within the prostate (27–30). In addition, the effects of metabolic disorders on BPH are not confined to local tissue changes; they also contribute to the systemic environment that promotes prostate growth. Obesity and insulin resistance induce systemic metabolic changes that exacerbate the local inflammatory and oxidative stress responses in the prostate (27, 29, 31). These systemic factors, in conjunction with local tissue changes, accelerate the development of BPH, creating a cycle where metabolic disturbances continuously fuel the progression of prostate enlargement. Therefore, the interaction between metabolic disorders and BPH not only accelerates the growth of prostate tissue locally but also promotes its systemic manifestation, further driving the progression of the disease.

#### 1.1.5 Epigenetic changes in BPH

Epigenetic modifications play a crucial role in the development and progression of BPH. Unlike genetic mutations, epigenetic changes do not alter the DNA sequence but affect gene expression through mechanisms such as DNA methylation, histone modifications, and non-coding RNAs (ncRNAs), influencing cellular functions like prostate epithelial cell proliferation, stromal remodeling, and immune responses. DNA hypermethylation is prevalent in BPH and affects genes, contributing to cell cycle dysregulation and fibrosis, whereas prostate cancer exhibits global hypomethylation (32). In addition, ncRNAs, particularly long non-coding RNAs (lncRNAs) and microRNAs (miRNAs), regulate inflammatory responses, androgen receptor signaling, and fibrosis, thereby promoting BPH progression (33–35). Histone modifications, including H3K27ac acetylation, enhance androgen receptor activation, while histone methylation is associated with increased fibrosis in BPH (36). These epigenetic changes create a complex regulatory network that sustains inflammation, proliferation, and stromal remodeling. Due to the significant role of epigenetic mechanisms in BPH pathophysiology, emerging therapeutic approaches are targeting these alterations, including DNA methylation inhibitors (37), histone deacetylase (HDAC) inhibitors (38), and micRNA-based (39) therapies to reduce fibrosis and slow BPH progression. Overall, epigenetic modifications contribute to BPH by altering gene expression, promoting inflammation, and inducing structural remodeling, distinguishing it from prostate cancer, which is characterized by genomic instability. Understanding and targeting these epigenetic alterations may provide novel strategies for slowing BPH progression and improving patient outcomes.

The pathological mechanisms of BPH involve multiple factors, with androgen action, chronic inflammation, immune cell infiltration, metabolic disorders, and epigenetic modifications playing crucial roles in the onset and progression of BPH. These mechanisms not only influence BPH through individual pathways but also interact with each other to drive the complex pathogenesis of the disease. Therefore, further in-depth research on the interplay between these mechanisms will provide a stronger theoretical foundation and clinical guidance for the prevention and treatment of BPH.

## 2 Drug treatment of BPH

BPH is a common condition in aging men, characterized by prostate enlargement, which leads to LUTS. LUTS can result from BOO due to prostate enlargement and bladder overactivity, leading to both voiding symptoms and storage symptoms. Historically, transurethral resection of the prostate (TURP) has been the gold standard surgical treatment for moderate-to-severe BPH cases. However, over the past few decades, there has been a shift from surgical intervention to medical management, particularly among elderly patients in the United States and globally (40). Current pharmacological treatments target different mechanisms of BPH progression and symptom relief. The choice of therapy depends on factors such as symptom severity, prostate size, patient comorbidities, and treatment preferences.

### 2.1 α-adrenergic receptor blockers in the treatment of BPH

BPH is a prevalent urological condition affecting aging men, often leading to LUTS and BOO. Among the pharmacological treatments available, α-adrenergic receptor blockers (α-blockers) are the most commonly prescribed due to their rapid symptom relief and strong clinical efficacy. These drugs have become the first-line treatment for BPH and are recommended in various international guidelines (41, 42). By targeting α1-adrenergic receptors, which are abundant in the prostate and bladder neck, α-blockers relax smooth muscle tissue, thereby reducing urinary flow resistance and improving voiding symptoms (43). The prostate and bladder neck contain a high density of α1-adrenergic receptors, which regulate smooth muscle contraction. When activated by adrenergic stimulation, these receptors increase smooth muscle tone, worsening urinary obstruction in BPH. α-blockers function by inhibiting these receptors, leading to smooth muscle relaxation in the prostate, urethra, and bladder neck. This mechanism reduces dynamic obstruction and improves urine flow without affecting prostate size. Due to their selective action on α1-receptors, these drugs provide rapid relief of LUTS, making them highly effective for symptom management (44). However, α-blockers do not shrink the prostate or prevent disease progression, which is why they are often used in combination with other medications such as 5α-reductase inhibitors for long-term management (44). Commonlyα-blockers are classified into non-selective α1-blockers and uroselective α1A-blockers, with newer drugs offering improved selectivity and fewer systemic side effects (**Table 1**).

**Table 1:** Commonly used α-blockers.

Drug name	Selectivity	Dosing	Advantages	Disadvantages
Tamsulosin (45) (Flomax)	Selective for α1A	Once daily	Less effect on blood pressure, fewer cardiovascular side effects	Higher incidence of retrograde ejaculation
Silodosin (46) (Rapaflo)	Highly selective for α1A	Once daily	Most uroselective, effective for LUTS	Higher risk of retrograde ejaculation
Alfuzosin (47) (Uroxatral)	Moderately selective	Once daily (ER formulation)	Fewer ejaculation problems	Mild dizziness, fatigue
Doxazosin (48) (Cardura)	Non-selective α1	Once daily	Reduces blood pressure, good for hypertensive patients	Can cause postural hypotension
Terazosin (49) (Hytrin)	Non-selective α1	Once daily	Effective in BPH + hypertension	May cause dizziness, fatigue

Numerous clinical studies have demonstrated the effectiveness of α-blockers in reducing LUTS severity and improving urinary flow in men with BPH. Research has shown that α-blockers can reduce the International Prostate Symptom Score (IPSS) by 30%-40% and increase maximum urinary flow rate (Qmax) by 20%-25%. Additionally, α-blockers have been found to significantly reduce the risk of acute urinary retention (AUR) and catheterization in patients with moderate-to-severe LUTS. These findings highlight the fast-acting benefits of α-blockers, as many patients experience symptom relief within days to weeks of starting treatment. However, while α-blockers are effective in improving urine flow, they do not alter prostate size, which is why they are often combined with 5α-reductase inhibitors (5α-RIs) for long-term disease management (45). Although α-blockers are generally well-tolerated, they are associated with several side effects, which can impact patient adherence. One of the most common side effects is orthostatic hypotension, especially in non-selective α-blockers like terazosin and doxazosin, which can cause dizziness, lightheadedness, and fainting. To minimize this risk, these medications are often taken at bedtime. Another significant concern is ejaculatory dysfunction, particularly with uroselective α-blockers like tamsulosin and silodosin, which can lead to retrograde ejaculation. Additionally, α-blockers, particularly tamsulosin, have been associated with Intraoperative Floppy Iris Syndrome (IFIS) during cataract surgery, which can complicate the procedure. Patients undergoing cataract surgery should inform their ophthalmologist if they are taking α-blockers (46). For patients with moderate-to-severe LUTS and large prostates (>40 mL), combination therapy with α-blockers and 5α-reductase inhibitors (5α-RIs) is often recommended. While α-blockers provide immediate symptom relief, 5α-RIs such as finasteride and dutasteride work by shrinking the prostate over time by inhibiting dihydrotestosterone (DHT) production (47–49). Clinical trials, such as the MTOPS (Medical Therapy of Prostatic Symptoms) trial, have demonstrated that combination therapy reduces BPH progression, lowers the risk of acute urinary retention, and decreases the need for surgical intervention (48). However, combination therapy is associated with higher rates of sexual dysfunction, requiring careful patient selection.The choice of α-blocker therapy should be tailored to the individual patient’s prostate size, LUTS severity, and comorbidities. For patients with mild-to-moderate LUTS and small prostates, α-blockers alone are sufficient. For those with larger prostates and progressive symptoms, combination therapy with 5α-reductase inhibitors is recommended. Patients with hypertension may benefit from non-selective α-blockers, while uroselective α-blockers like tamsulosin and silodosin are preferred for those at risk of hypotension. Additionally, patients with erectile dysfunction (ED) may benefit from PDE5-Is, such as tadalafil, rather than α-blockers.

### 2.2 Phosphodiesterase-5 inhibitors in the treatment of BPH

Phosphodiesterase-5 (PDE - 5) inhibitors, originally designed for the treatment of erectile dysfunction (ED), have emerged as a promising therapeutic option for managing LUTS in men with BPH (50). These drugs exert their effects by enhancing smooth muscle relaxation and improving vascular perfusion, thereby alleviating symptoms associated with urinary obstruction. Among PDE - 5 inhibitors, tadalafil is the only FDA-approved drug for the treatment of BPH-related LUTS (51). Studies have shown that PDE - 5 inhibitors not only improve urinary symptoms but also simultaneously address erectile dysfunction, making them particularly beneficial for men with comorbid BPH and ED (52). Phosphodiesterase-5 (PDE - 5) inhibitors exert their therapeutic effects by inhibiting the enzyme phosphodiesterase-5, which degrades cyclic guanosine monophosphate (cGMP) in smooth muscle cells. By increasing cGMP levels, these drugs promote the relaxation of smooth muscle in the bladder neck, prostate, and urethra, leading to improved urinary flow and relief from LUTS such as weak stream and hesitancy (50, 53). Additionally, PDE - 5 inhibitors enhance vascular perfusion in the prostate and bladder, potentially reducing inflammation and oxidative stress, which are implicated in the progression of BPH. While PDE - 5 inhibitors do not directly reduce prostate size, they alleviate smooth muscle tension, making them effective for managing LUTS (54). Furthermore, these drugs may have anti-inflammatory and anti-oxidative effects that protect prostate tissue, potentially slowing the progression of BPH. Although PDE - 5 inhibitors are not used to shrink the prostate, their ability to relieve urinary symptoms and improve erectile dysfunction, particularly when combined with α-blockers, highlights their significant role in BPH treatment. Commonly Phosphodiesterase-5 (PDE - 5) Inhibitors are classified into Tadalafil, Sildenafil, Vardenafil and Avanafil (**Table 2**). Clinical studies have consistently demonstrated the efficacy of PDE - 5 inhibitors in improving LUTS and erectile function. Tadalafil (5 mg daily) has been shown to significantly reduce IPSS and enhance International Index of Erectile Function (IIEF) scores in men with BPH. However, some studies have reported that tadalafil does not significantly improve urodynamic parameters such as maximum urinary flow rate (Qmax), bladder capacity, or detrusor pressure (55). Sildenafil, another PDE - 5 inhibitor, has also been found to improve IPSS and erectile function, with some studies suggesting superior effects on reducing post-void residual volume (PVR) and improving quality of life compared to tadalafil. These findings highlight the potential of PDE - 5 inhibitors to provide meaningful symptomatic relief in BPH-related LUTS (56). PDE - 5 inhibitors are often used in combination with other medications, such as α-blockers, to enhance therapeutic outcomes. This combination leverages the immediate symptom relief provided by α-blockers and the dual action of PDE - 5 inhibitors on LUTS and erectile function. Studies have demonstrated that this approach is particularly effective for men with moderate-to-severe LUTS and concurrent ED, offering greater symptom improvement than monotherapy alone (57).

**Table 2:** Commonly used phosphodiesterase-5 (PDE-5) inhibitors.

Drug name	Primary use	Dosage	Key advantages	Disadvantages
Tadalafil (59)(Cialis)	BPH and ED	5 mg daily (for BPH) or 10-20 mg as needed (for ED)	FDA-approved for BPH and ED; improves IPSS and IIEF; long half-life allows once-daily dosing	No effect on prostate size; may cause headaches, flushing
Sildenafil (62) (Viagra)	ED (off-label for BPH)	50-100 mg as needed	Improves IPSS and ED; may reduce PVR volume	Not FDA-approved for BPH; may cause headaches, flushing
Vardenafil (63) (Levitra)	ED (off-label for BPH)	10 mg as needed	Similar efficacy to sildenafil; faster onset of action	Not FDA-approved for BPH; side effects similar to sildenafil
Avanafil (64)(Stendra)	ED (off-label for BPH)	50-100 mg as needed	Fast onset (15-30 minutes); fewer side effects compared to others	Not FDA-approved for BPH; may still cause mild headaches

In a study, 60 men with BPH-related LUTS were randomly assigned to one of three groups: sildenafil (25 mg) alone (n = 20), tamsulosin (0.4 mg daily) alone (n = 20), or a combination of both (n = 20), for 8 weeks. Significant improvements were observed across all groups in terms of IPSS, maximum urinary flow rate (Qmax), post-void residual (PVR) volume, Sexual Health Inventory for Male (SHIM) scores, and IIEF questions 3 and 4. Symptom relief was most pronounced in the combination therapy group (40.1%) and the tamsulosin-only group (36.2%), compared to the sildenafil-only group (28.2%), with a p-value of less than 0.001. The tamsulosin-only and combination groups also showed greater improvement in Qmax and PVR volume than the sildenafil-only group. In terms of sexual health, SHIM scores improved substantially more in both the sildenafil-only (65%) and combination groups (67.4%) than in the tamsulosin-only group (12.4%; p < 0.001), and IIEF scores were also significantly higher in the sildenafil and combination therapy groups (58). This study concluded that combining tamsulosin with sildenafil did not offer superior benefits over tamsulosin alone in terms of relieving voiding symptoms. PDE5i represent a valuable pharmacological option for men with LUTS associated with BPH, especially those with concurrent erectile dysfunction. By promoting smooth muscle relaxation and enhancing vascular perfusion, these drugs provide significant symptomatic relief and improve quality of life. While they do not reduce prostate size or alter disease progression, their ability to address multiple symptoms makes them a cornerstone of BPH management for specific patient populations. Future research may focus on optimizing their use in combination therapies and expanding their indications for broader application.

### 2.3 5‐Alpha reductase inhibitors in the treatment of BPH

5α-reductase inhibitors (5-ARIs), such as finasteride and dutasteride, are among the most commonly prescribed medications for managing BPH. These drugs primarily work by inhibiting the enzyme 5α-reductase, which plays a central role in the conversion of testosterone into dihydrotestosterone (DHT), the androgen responsible for stimulating prostate growth. DHT is a powerful androgen that drives both the growth of prostate tissue and the associated LUTS in BPH (59). 5α-reductase inhibitors (5-ARIs), such as finasteride and dutasteride, primarily target the enzyme 5α-reductase, which plays a key role in the conversion of testosterone into dihydrotestosterone (DHT), a potent androgen responsible for prostate growth (60). The enzyme 5α-reductase exists in two isoforms: Type 1,This isoform is predominantly found in non-prostatic tissues, such as the skin and liver. It contributes to the formation of DHT in the skin and other tissues but has a lesser role in the prostate. Type 2, This isoform is the predominant form in the prostate, accounting for the majority of DHT production in prostate tissue. Type 2 5α-reductase is the primary enzyme involved in the prostate growth seen in BPH (61–64). The role of 5-ARIs is to inhibit the activity of 5α-reductase, thereby blocking the conversion of testosterone into DHT. As a result, these medications decrease the levels of DHT in the prostate, which is crucial for reducing prostate growth. With lower DHT levels, the prostate shrinks, leading to a reduction in the compression of the urethra and an improvement in urinary flow. This results in a significant alleviation of LUTS, such as frequent urination, urgency, and nocturia, which are associated with BPH (65). Finasteride selectively inhibits 5α-reductase type 2. By targeting this specific isoform, finasteride reduces DHT levels in the prostate, which is the primary site of action for managing BPH-related symptoms (66). Dutasteride, on the other hand, inhibits both type 1 and type 2 5α-reductase isoforms, providing a broader and more comprehensive suppression of DHT production. This dual inhibition allows dutasteride to reduce DHT levels in both the prostate and other tissues (such as the skin and liver), which may provide additional benefits in terms of reducing overall prostate size and symptom relief (67). commonly used 5-alpha reductase inhibitors and their associated advantages and disadvantages (**Table 3**).

**Table 3:** Commonly used 5‐alpha reductase inhibitors.

Drug name	Primary use	Dosage	Advantages	Disadvantages
Finasteride (75)	BPH treatment, male pattern baldness	5 mg daily (for BPH) or 1 mg daily (for hair loss)	Well-established safety profile; effective in reducing prostate size and improving urinary flow	May cause sexual side effects such as decreased libido and erectile dysfunction
Dutasteride (76)	BPH treatment	0.5 mg daily	Inhibits both type 1 and type 2 5α-reductase; potentially more effective than finasteride in reducing prostate size	Higher risk of sexual side effects; more expensive than finasteride
Epristeride (72)	BPH treatment	5 mg daily (for BPH)	Selective inhibition of 5α-reductase type 2; lower side-effect profile compared to other 5-ARIs	Less commonly used; fewer long-term studies available

By inhibiting these enzymes, 5-ARIs reduce DHT-mediated prostate growth, which not only reduces prostate size but also improves urinary flow and relieves symptoms of BPH. Over time, the reduction in prostate size helps to decrease urinary retention, improve Qmax (maximum urinary flow rate), and lower the need for surgical interventions like TURP. One of the key studies, the Proscar Long-Term Efficacy and Safety Study (PLESS), demonstrated that finasteride reduced the need for surgical intervention by 55%, and the risk of acute urinary retention decreased by 57%. Additionally, IPSS, a measure of symptom severity, showed an average reduction of 3.3 points, reflecting significant symptom relief. However, finasteride is associated with certain sexual side effects, such as reduced libido and erectile dysfunction, which may limit its use in some patients. Comparing dutasteride to a placebo showed a 23% reduction in prostate volume and a 17% improvement in urinary flow rate (Qmax). Additionally, dutasteride has been shown to provide greater improvements in IPSS and QoL scores in patients with more severe BPH symptoms. Like finasteride, dutasteride is associated with sexual side effects such as reduced libido, erectile dysfunction, and ejaculatory disorders, though the dual inhibition may provide better symptom relief in patients who require a stronger DHT reduction (68).

### 2.4 Anticholinergics drugs in the treatment of BPH

Anticholinergic drugs are widely used in the treatment of bladder overactivity, which is a common symptom in patients with BPH. These medications work by targeting the muscarinic receptors (specifically M2 and M3) on the detrusor muscle of the bladder. The activation of these receptors by acetylcholine (ACh) causes contraction of the bladder muscle, leading to urinary urgency and frequency (69). Anticholinergic drugs block the muscarinic receptors, reducing the effects of acetylcholine, and subsequently relaxing the detrusor muscle. This results in reduced urinary urgency, increased bladder storage capacity, and overall improvement in storage symptoms associated with BPH (70). Anticholinergic drugs, such as oxybutynin, tolterodine, and solifenacin, are commonly used to treat bladder overactivity, a frequent symptom of BPH. These medications work by targeting the muscarinic receptors on the detrusor muscle of the bladder, specifically the M2 and M3 subtypes (71). When acetylcholine (ACh) binds to these receptors, it triggers contraction of the detrusor muscle, leading to symptoms like urinary urgency, frequency, and nocturia. By blocking the effects of acetylcholine, anticholinergics reduce detrusor muscle contraction, improving bladder storage, alleviating urgency, and decreasing frequency and nocturia. While these medications are effective for managing bladder overactivity, they have limited efficacy when used alone for voiding symptoms such as weak stream, incomplete bladder emptying, or difficulty starting urination, which are typically caused by prostate enlargement and mechanical obstruction. Therefore, anticholinergic drugs are most beneficial for patients with storage symptoms, but less so for those with primarily voiding difficulties. Given these limitations, anticholinergics are often combined with other BPH treatments, such as α-blockers (e.g., tamsulosin) to relax smooth muscle in the prostate and bladder neck, or 5-ARIs (e.g., finasteride and dutasteride) to shrink the prostate, offering better overall symptom relief and improved quality of life for patients suffering from both storage and voiding symptoms (72). The commonly used anticholinergic drugs in the treatment of BPH(**Table 4**) (73).

**Table 4:** Commonly used anticholinergics.

Drug name	Primary use	Dosage	Advantages	Disadvantages
Oxybutynin	Treatment for bladder overactivity in BPH patients	5 mg once daily (or adjusted based on patient response)	- Effectively relieves urgency, frequency, and nocturia. - Improves bladder storage capacity. - Non-invasive, oral treatment option for BPH symptoms.	- Limited efficacy for voiding symptoms. - Side effects like dry mouth, constipation, blurred vision, and urinary retention. - Potential cognitive risks in elderly patients.
Tolterodine	Treatment for bladder overactivity in BPH patients	2 mg twice daily (may adjust based on patient response)	- Similar to oxybutynin in reducing urgency and frequency. - Better tolerated in some patients compared to oxybutynin.	- Less effective for voiding symptoms. - May cause dry mouth and constipation. - Cognitive concerns for elderly patients.
Solifenacin	Treatment for bladder overactivity in BPH patients	5 mg once daily (or adjusted based on patient response)	- Effective for urgency, frequency, and nocturia. - Less anticholinergic side effects compared to older drugs.	- Limited effectiveness for voiding symptoms. - Side effects may still include dry mouth, constipation, and vision issues. - Cognitive decline risk for older adults.

Anticholinergic drugs are valuable in managing bladder overactivity in patients with BPH. They provide significant benefits for alleviating urgency, frequency, and nocturia but have limited efficacy when it comes to voiding symptoms. Due to their potential cognitive side effects, particularly in the elderly, their use should be approached with caution, especially for long-term treatment. Combining anticholinergic drugs with α-blockers or 5α-reductase inhibitors (5-ARIs) often offers better overall symptom relief for BPH patients.

### 2.5 β-3 Agonists

Beta-3 adrenergic receptors are primarily located on the detrusor smooth muscle of the bladder, where their activation plays a crucial role in mediating bladder relaxation. When stimulated by beta-3 adrenergic agonists, these receptors facilitate detrusor muscle relaxation, leading to increased bladder capacity, reduced voiding frequency, and improved storage LUTS. Unlike anticholinergic drugs, which target muscarinic receptors to reduce bladder overactivity, beta-3 agonists achieve similar therapeutic benefits without causing significant anticholinergic side effects, such as dry mouth, constipation, and urinary retention (72). Several studies have evaluated the effectiveness of beta-3 adrenergic agonists, particularly mirabegron, in treating LUTS associated with BPH (74). Clinical trials suggest that mirabegron improves storage symptoms, such as urgency, frequency, and nocturia, without significantly affecting voiding symptoms like urinary flow rate (Qmax) or post-void residual volume (PVR) (75). However, it is essential to note that most early studies on beta-3 agonists focused on patients with overactive bladder (OAB), rather than specifically on men with BPH. One of the most notable clinical studies was conducted by Nitti et al., in which mirabegron (50 mg and 100 mg) was compared to placebo in 176 men diagnosed with LUTS and BOO. The results showed: Significant improvements in the mean number of micturitions per day and urgency episodes in the mirabegron group compared to placebo. No significant differences in IPSS or adverse events between mirabegron and placebo.

No significant difference in detrusor pressure and maximum urinary flow rate (Qmax), suggesting that mirabegron does not worsen urinary obstruction (76). Here is a structured table format summarizing Beta-3 Agonists in the treatment of BPH (**Table 5**).

**Table 5:** Commonly used anticholinergics.

Drug name	Primary use	Dosage	Advantages	disadvantages
Mirabegron (86)	Treatment of storage symptoms (urgency, frequency, nocturia) in LUTS associated with BPH	25-50 mg once daily	- Improves bladder storage symptoms (urgency, frequency, nocturia) - Lower risk of dry mouth, constipation, and urinary retention compared to anticholinergics - No significant impact on cognitive function	- Limited efficacy in improving voiding symptoms (e.g., weak stream, incomplete bladder emptying) - Not effective for reducing prostate size - Potential for hypertension in some patients
Vibegron (87)	Treatment of storage LUTS in BPH patients with BOO	50 mg once daily	- Effective alternative to mirabegron - Fewer side effects than anticholinergics - Less risk of urinary retention	- Limited research in BPH patients - Not effective in improving voiding symptoms

Beta-3 adrenergic agonists, such as mirabegron, offer a promising alternative to anticholinergics for treating BPH-related LUTS, particularly in storage symptoms like urgency, frequency, and nocturia. Compared to anticholinergics, they provide similar efficacy but with fewer side effects, such as dry mouth, constipation, and urinary retention. However, these drugs have limited impact on voiding symptoms and prostate enlargement, making them less effective as monotherapy in men with significant BOO. As a result, they are best used in combination therapy with alpha-blockers for comprehensive symptom relief in BPH-associated LUTS.

### 2.6 Combination therapy: the future of BPH treatment

#### 2.6.1 α-blockers + 5-ARIs

The combination of α-blockers and 5-ARIs is considered the gold standard for treating patients with large prostates (>40 mL) who experience significant BOO. α-blockers such as tamsulosin and alfuzosin provide rapid symptom relief by relaxing the smooth muscle in the prostate and bladder neck, which improves urinary flow. However, these drugs do not reduce prostate size. On the other hand, 5-ARIs such as finasteride and dutasteride target hormonal pathways by inhibiting 5α-reductase, the enzyme responsible for converting testosterone to dihydrotestosterone (DHT), a key hormone driving prostate growth. This dual approach provides both immediate symptom relief (via α-blockers) and long-term reduction in prostate size (via 5-ARIs). The combination of α-blockers and 5-ARIs has been extensively evaluated in clinical trials, with two major randomized controlled studies—the Medical Therapy of Prostatic Symptoms (MTOPS) trial and the Combination of Tamsulosin and Finasteride (CombAT) trial)—providing strong evidence for its effectiveness. In particular, McConnell J D et al. finished the trial, which included 3,000 men randomized to receive either a placebo, doxazosin, finasteride, or a combination of both, found that combination therapy reduced the risk of BPH progression by 66%. This reduction was significantly greater than what was observed with monotherapy, where doxazosin alone decreased risk by 39% and finasteride alone by 34%. These findings underscore the superior efficacy of combination therapy in not only alleviating symptoms but also preventing disease progression compared to either medication used alone (68). Similarly, the CombAT Trial (2010) confirmed that a combination of Dutasteride and Tamsulosin was more effective than monotherapy in reducing AUR and the need for surgery (77). However, sexual side effects such as erectile dysfunction, decreased libido, and retrograde ejaculation are more common in combination therapy, requiring careful patient selection.

#### 2.6.2 α-blockers + phosphodiesterase-5 inhibitors

The combination of α-blockers and phosphodiesterase-5 (PDE5) inhibitors is an effective treatment approach for men who suffer from both BPH and erectile dysfunction (ED). This therapy offers a dual benefit by improving urinary symptoms and sexual function simultaneously. α-blockers such as Tamsulosin and Silodosin work by relaxing smooth muscle in the prostate and bladder neck, leading to improved urine flow and relief from LUTS. On the other hand, PDE5 inhibitors like Tadalafil and Sildenafil enhance nitric oxide (NO) signaling, which improves vascular perfusion in the prostate and bladder, leading to smooth muscle relaxation and symptom relief in LUTS patients. Clinical evidence from trials such as the LUTS-ED Trial (2011) has demonstrated that Tadalafil (5 mg daily) significantly improved IPSS scores and Qmax (maximum urine flow rate) in men experiencing both BPH and ED (56). Additionally, the REACT Trial (2016) confirmed that the combination of Tadalafil and Tamsulosin was more effective than either drug alone in reducing urinary symptoms and enhancing erectile function (78). This combination therapy is well-tolerated and presents fewer sexual side effects, particularly when compared to α-blockers alone, which are often associated with retrograde ejaculation. Despite its benefits, this therapy also has some limitations. Unlike 5-ARIs, PDE5 inhibitors do not shrink the prostate, making them less suitable for patients with very large prostates. Additionally, these drugs may cause mild systemic side effects such as headaches, flushing, and nasal congestion. A significant contraindication for PDE5 inhibitors is their interaction with nitrates, which can lead to severe hypotension, making them unsuitable for patients with cardiovascular diseases requiring nitrate therapy. Overall, α-blockers combined with PDE5 inhibitors are an excellent option for men with moderate prostate enlargement and concurrent ED, providing immediate symptom relief and improving overall quality of life.

#### 2.6.3 β3 agonists + α-blockers

The combination of β3 agonists and α-blockers is an effective therapeutic approach for BPH patients with overactive bladder (OAB) features and predominant storage symptoms, such as urgency, frequency, and nocturia, while still addressing some voiding symptoms. α-blockers like Tamsulosin work by relaxing the smooth muscle in the prostate and bladder neck, helping to improve urinary flow and reduce voiding difficulties. Meanwhile, β3 agonists such as Mirabegron and Vibegron target β3 adrenergic receptors on the detrusor muscle, allowing for bladder relaxation, which increases bladder capacity and reduces storage symptoms. Clinical trials support the efficacy of this combination therapy. The MATCH Study (2022) demonstrated that Mirabegron + Tamsulosin was superior to Tamsulosin alone in reducing storage symptoms, without increasing the risk of urinary retention, making it a safer alternative to anticholinergics (79). Additionally, the Vibegron Study (2023) found that adding Vibegron to α-blocker therapy resulted in better symptom control for patients experiencing both BPH and OAB symptoms. The advantages of this combination therapy include its ability to improve both storage and voiding symptoms, while posing less risk of urinary retention compared to anticholinergic drugs (80). It is also well-tolerated, with a lower incidence of dry mouth and constipation, making it preferable for older patients or those prone to cognitive side effects from anticholinergics. However, this therapy does not shrink the prostate, making it less effective for men with significantly enlarged prostates. Additionally, its impact on moderate to severe BOO is limited, and some patients may experience mild hypertension as a side effect of β3 agonists. Despite these limitations, this combination therapy offers a promising alternative for BPH patients experiencing both storage and voiding symptoms who may not tolerate traditional anticholinergic treatments.

#### 2.6.4 Anticholinergics + α-blockers

The combination of anticholinergics and α-blockers is particularly beneficial for men with severe storage symptoms, such as urgency, frequency, and nocturia, although caution is required in patients with BOO due to the potential risk of urinary retention. α-blockers, such as Tamsulosin and Alfuzosin, help reduce voiding resistance in the prostate and bladder neck, facilitating urine flow. On the other hand, anticholinergic drugs, including Oxybutynin, Solifenacin, and Tolterodine, work by blocking muscarinic receptors in the bladder, reducing involuntary bladder contractions, and thereby alleviating urgency, frequency, and nocturia. Clinical evidence supports this approach. One Study found that Solifenacin + Tamsulosin provided better symptom relief in men with severe urgency and frequency compared to α-blocker monotherapy (79). Similarly, another Study demonstrated that Oxybutynin + Tamsulosin effectively controlled storage symptoms but also increased the risk of urinary retention in men with significant BOO, emphasizing the need for careful patient selection (81). This combination offers several advantages, including its high effectiveness in treating severe storage symptoms, substantial improvement in urgency, frequency, and nocturia, and better outcomes for men with small prostates but significant LUTS. However, there are notable limitations as well, such as an increased risk of urinary retention, particularly in BOO patients, potential cognitive side effects (especially in elderly patients), and common adverse effects like dry mouth and constipation. Due to these factors, anticholinergic therapy is generally reserved for men whose predominant symptoms involve bladder overactivity rather than voiding dysfunction.

### 2.7 Conclusion

Combination therapy has revolutionized BPH treatment, providing targeted symptom relief while reducing disease progression. The choice of combination depends on patient-specific factors such as prostate size, predominant symptoms (voiding vs. storage), and comorbidities (e.g., ED, OAB) (**Table 6**).

**Table 6:** Comprehensive table of combination therapies for BPH.

Combination Therapy	α-Blockers + 5-ARIs (92–95)	α-Blockers + PDE5 Inhibitors (54, 93, 96)	β3 Agonists + α-Blockers (93, 97–99)	Anticholinergics + α-Blockers (93, 100, 101)
Drug Names	Tamsulosin, Alfuzosin + Finasteride, Dutasteride	Tamsulosin + sildenafil, tadalafil	Mirabegron + Tamsulosin, Doxazosin	Oxybutynin + Tamsulosin, Doxazosin
Best For	Patients with large prostate volumes (>40 mL), high acute urinary retention risk	Patients with BPH and Erectile Dysfunction (ED)	Patients with BPH and storage symptoms (urgency, frequency)	Patients with severe storage symptoms (urgency, frequency, nocturia), especially with Overactive Bladder (OAB)
Primary Benefit	Reduces prostate size, lowers surgery risk, improves symptoms	Improves LUTS and sexual function	Increases bladder capacity, improves symptom control, reduces storage symptoms	Reduces urgency, frequency, nocturia (with caution in BOO)
Onset of Action	α-Blockers: Immediate<br>5-ARIs: 3-6 months	2-4 weeks	2-6 weeks	1-2 weeks
Risk of Urinary Retention	Low	Low	Low	High (especially in BOO)
Common Side Effects	Erectile dysfunction (ED), low libido, postural hypotension	Headache, flushing, nasal congestion, hypotension	Mild hypertension, nausea, dizziness	Dry mouth, constipation, cognitive impairment (elderly)
Drug Interactions	Possible increased risk of hypotension with antihypertensives	Possible increased risk of hypotension with α-Blockers	Possible enhanced hypotensive effect with α-Blockers	Possible increased risk of hypotension with α-Blockers
Long-term Efficacy	Significant, especially with long-term use	Durable, particularly with continuous therapy	Durable, especially with long-term use	Short-term effectiveness, long-term use requires caution
Patient Quality of Life Improvement	Significant improvement, enhanced quality of life	Significant improvement in sexual health and overall well-being	Significant improvement in storage symptoms and quality of life	Improvement possible, but side effects need attention

## 3 Surgical treatment of BPH

Surgical interventions remain an important treatment option for patients who experience inadequate relief from medications or have severe complications like acute urinary retention, recurrent infections, or significant prostate enlargement. Although many patients find symptom relief through medications, some may face side effects such as ineffective symptom control or adverse reactions, such as urinary retention or sexual dysfunction. For those who do not respond to medications or experience unacceptable side effects (such as urinary retention), surgical treatment becomes a critical option (82). Procedures like TURP, Laser Surgery, and Prostatectomy are common. While surgery offers significant benefits in terms of symptom relief, it is not without its challenges. Catheterization, required in cases of acute urinary retention, often becomes a necessary short-term or long-term solution for some individuals. Patients may find the psychological, social, and physical implications of catheter use uncomfortable or even distressing, especially considering the impact on quality of life. Some patients may perceive catheter insertion as more disruptive to their lives than undergoing surgery itself (83).

**Figure 1:**
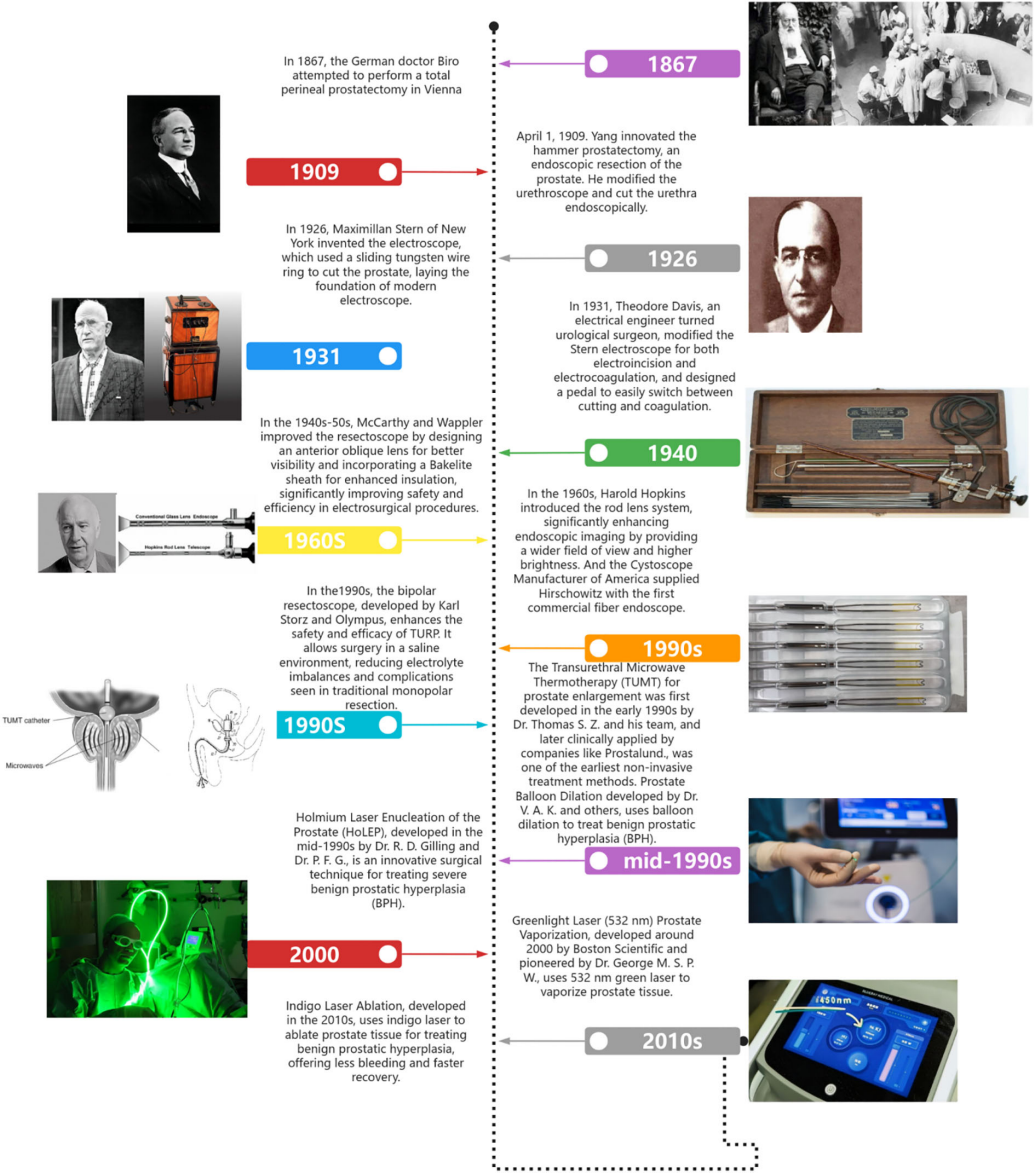
The history of the development of surgery for BPH.

The surgical treatment of BPH has undergone significant development over the past centuries, evolving from open prostatectomy in the 19th century to TURP in the mid-20th century. With continuous advancements in surgical instruments, such as more precise resectoscopes and electrocautery techniques, as well as the introduction of laser technologies (e.g., laser prostatectomy and laser vaporization), the safety, precision, and postoperative recovery speed of these procedures have greatly improved, significantly reducing the incidence of postoperative complications and gradually becoming the standard surgical approach for treating BPH (**Figure 1**).

### 3.1 Introduction of TURP

The treatment of BPH, has evolved significantly over time, with substantial advancements in both surgical techniques and medical management. In the early days, prostate disease treatments were limited to rudimentary and invasive surgeries, which often carried high risks of complications such as infection and bleeding. However, the introduction of more refined techniques, such as TURP, revolutionized the field by offering less invasive solutions. This shift has greatly improved patient outcomes, allowing for more effective symptom relief with shorter recovery times and a reduction in post-operative risks.

Before the 20th century, the treatment of BPH was quite limited, and most options were highly invasive, often with severe risks. In the 1800s, open prostatectomy, a procedure where the prostate was removed through abdominal incisions, was one of the few options for treating BPH. These procedures had high complication rates, including infection, bleeding, and long recovery periods. The lack of precision and the invasive nature of these early surgeries made them dangerous, and many patients had poor outcomes (84). In 1926, the first step toward TURP was taken when the cystoscope was combined with the tubular punch. This advancement allowed for a more direct approach to prostate tissue removal, an important development in the history of BPH treatments. The tungsten loop was then introduced to enable more precise tissue resection. Prior to these innovations, surgical procedures were blind, relying on a less accurate approach to tissue removal. The introduction of these new tools in the early 1930s paved the way for more refined surgical techniques. In 1932, McCarthy developed a combination of instruments that incorporated an oblique lens. This made it possible to perform resection under direct vision, significantly improving the surgeon’s ability to visualize the prostate tissue and ensuring that tissue removal was done more accurately. This was a major advancement from the previous blind procedures (85, 86). The 1960s-1970s saw further progress in the field with the advent of fiber optic lighting systems. These systems greatly enhanced endoscopic visualization, allowing surgeons to view the surgical site with greater clarity. In 1976, the rod-lens system was developed by Hopkins, improving the amount of light transmitted through the lens. This improvement in light transmission enabled surgeons to have an even better view during the procedure, enhancing the accuracy and safety of the resection process (87, 88). These advancements contributed significantly to TURP becoming the gold standard in the treatment of BPH by the mid-20th century. Over time, the technique continued to evolve, with improvements in surgical instruments and the development of minimally invasive options, which further refined prostate surgery and minimized patient recovery time. In the 1980s, TURP was one of the most frequently performed surgeries, ranking second only to phacoemulsification, which is a procedure for cataract treatment. During this period, TURP was the gold standard for treating BPH, as it provided effective symptom relief for patients suffering from urinary obstruction (89). However, pharmacological therapies and minimally invasive surgical techniques began to emerge and gain traction in the 1990s. These advancements contributed to a gradual decline in the number of TURP procedures performed. Between 1980 and 1991, the rate of TURP procedures decreased from 268.3 per 100,000 people to 229.2 per 100,000 people. By 1994, this figure had dropped further to 131.3 per 100,000 people. This decline was attributed to the introduction of new medications, such as α-blockers and 5-ARIs, which offered less invasive alternatives for symptom management, as well as the development of minimally invasive surgical options like laser prostatectomy and TUNA (Transurethral Needle Ablation). In 2005, TURP accounted for only 39% of the BPH procedures performed, a significant reduction from the 81% of BPH procedures in 1999. This decrease reflected the growing popularity and use of alternative therapies, including medications and non-invasive techniques. Despite this shift, TURP remains a reliable option for patients with large prostates or those who are not responsive to pharmacological treatments (90).

The resection technique in TURP follows a structured approach to ensure precise tissue removal while minimizing complications. The procedure begins with the identification of key anatomical landmarks, particularly the verumontanum, a ridge-like structure in the posterior urethra that serves as a critical marker to prevent damage to the external urinary sphincter, thereby reducing the risk of postoperative incontinence. The bladder neck, which marks the junction between the bladder and prostate, is also carefully identified to maintain proper orientation and avoid excessive tissue removal, which could lead to bladder dysfunction. Once the landmarks are clearly visualized using the resectoscope, the lobe-by-lobe resection process begins. The surgeon first creates resection trenches at the 5 o’clock and 7 o’clock positions using an electrosurgical loop. These trenches help define the capsular plane, guiding precise resection. The median lobe, if present, is typically resected first, as its enlargement can significantly obstruct the bladder outlet. Removing it creates an open funnel-shaped channel, which improves urinary flow. This is followed by the lateral lobe resection, where the left and right lobes are removed using a proximal-to-distal approach in small sweeping motions. This ensures a gradual and controlled removal of prostate tissue while preserving the integrity of the surrounding structures. The final phase of tissue removal involves the apical resection, where any remaining tissue near the apex of the prostate is carefully removed. Special attention is given to the verumontanum region to avoid damaging the external sphincter, which is crucial for maintaining urinary continence. After the resection phase, the excised prostate tissue must be evacuated from the bladder. Small tissue fragments naturally flow into the bladder due to continuous irrigation, while larger pieces that remain are manually pushed into the bladder using the resectoscope loop. The surgeon then uses evacuation techniques to remove the resected tissue. This is typically achieved using an Ellik evacuator, a bulb-like suction device that allows the surgeon to manually flush out and remove tissue fragments. In modern procedures, a morcellation system may be used, which helps break down larger tissue pieces into smaller fragments for faster and more efficient evacuation. Once tissue removal is complete, the resected cavity is re-inspected to ensure no residual tissue remains, the capsular plane is intact, and hemostasis has been achieved. The final step of the procedure involves hemostasis and catheterization. The surgeon carefully revisits the resected area, using the electrosurgical loop to coagulate bleeding vessels, effectively controlling any active bleeding. A three-way Foley catheter (22 – 24 French) is inserted into the bladder to allow continuous bladder irrigation (CBI). This irrigation process helps prevent clot formation, ensuring a clear urinary pathway while the healing process begins. The catheter remains in place for 24 – 48 hours, depending on the patient’s recovery status and urine clarity. Following TURP, patients typically remain in the hospital for 1 – 2 days for monitoring. The catheter is removed once hematuria subsides, and urine flow is adequate. Most patients experience symptom relief within 2 – 4 weeks, although complete recovery and stabilization of urinary function may take several months. TURP remains the gold standard for treating moderate-to-severe BPH, offering effective symptom relief, improved urinary flow, and reduced risk of AUR. Its advantages include precise tissue removal, minimal damage to surrounding structures, and effective bleeding control through electrosurgical coagulation. However, the procedure carries potential risks, including postoperative bleeding, retrograde ejaculation, temporary urinary incontinence, and in rare cases, TUR syndrome due to excessive fluid absorption. Despite these risks, TURP continues to be the most commonly performed surgical intervention for BPH, with ongoing improvements such as bipolar TURP, which reduces complications and improves patient safety (91, 92).

Although TURP remains the gold standard for the treatment of moderate-to-severe BPH, it is associated with certain risks, including retrograde ejaculation, urinary incontinence, and bleeding complications. As a result, careful patient selection and postoperative monitoring are crucial to optimizing outcomes. Despite the emergence of MISTs, TURP continues to be the benchmark against which newer BPH treatments are compared, offering durable, well-established, and clinically proven results. The future of TURP is being shaped by technological advancements aimed at enhancing safety, surgical precision, and functional outcomes. Bipolar TURP and plasma vaporization TURP are key innovations that have significantly reduced the risk of bleeding and TUR syndrome by utilizing saline irrigation instead of glycine-based solutions, making the procedure safer, particularly for patients on anticoagulation therapy. Additionally, the integration of robotic-assisted TURP and AI-driven image-guided techniques is expected to improve surgical accuracy, reduce inter-operator variability, and optimize resection strategies through real-time 3D imaging and AI-based decision support. Further hybrid surgical approaches, combining TURP with laser technologies such as Holep or GreenLight laser therapy, are emerging to enhance efficacy, reduce recovery time, and lower complication rates. Moreover, artificial intelligence-driven patient selection models are expected to play a pivotal role in the future of TURP by utilizing machine learning algorithms to identify ideal candidates for the procedure based on prostate volume, symptom severity, and comorbidities. This could enable more personalized treatment strategies, optimizing patient outcomes while minimizing risks. Given that ejaculatory dysfunction remains a significant concern post-TURP, innovative techniques such as ejaculatory hood-sparing TURP are being explored to preserve sexual function while maintaining the therapeutic benefits of the procedure. As new technologies continue to evolve, TURP remains the cornerstone of BPH management, with ongoing refinements making it safer, more precise, and more patient-tailored in the face of increasing competition from minimally invasive alternatives. In conclusion, while MISTs continue to gain popularity, TURP remains an indispensable treatment for BPH due to its proven long-term efficacy. With continuous advancements in robotics, imaging, artificial intelligence, and regenerative medicine, TURP is expected to evolve into a more sophisticated, personalized, and minimally invasive procedure, further solidifying its role in the future of prostatic surgery.

### 3.2 Introduction of HoLEP

HoLEP is a surgical procedure that utilizes holmium laser to precisely enucleate and remove hyperplastic prostate tissue while preserving the prostatic capsule. The procedure involves the use of a high-powered Holmium: YAG laser to enucleate and remove excessive prostatic adenoma, which causes urinary obstruction. Unlike TURP, which resects prostate tissue in small portions, HoLEP completely removes the obstructive adenoma, mimicking the open simple prostatectomy (OSP) technique but without the need for a large surgical incision. The enucleated prostate tissue is then fragmented within the bladder using morcellation and extracted. This approach allows for greater precision, minimal bleeding, and a significantly lower risk of complications compared to traditional BPH surgeries (93). Since its introduction in the 1990s, HoLEP has undergone continuous refinement and technological improvements, leading to its increasing adoption as the preferred surgical treatment for large prostates (≥80–100 mL), particularly in patients who are at higher risk of bleeding or require anticoagulation therapy (94). Clinical studies have shown that HoLEP provides greater symptom relief, lower rates of retreatment, and fewer complications compared to TURP, making it a gold-standard option for large prostates. One of the key advantages of HoLEP is its ability to significantly reduce prostate size while preserving bladder function, leading to improved urinary flow, reduced post-void residual volume, and sustained long-term benefits (93). Additionally, HoLEP eliminates the risk of TUR syndrome, a serious complication associated with excessive fluid absorption during traditional TURP procedures. Despite its steep learning curve, the growing adoption of HoLEP worldwide is a testament to its safety, efficacy, and long-term benefits. As more urologists receive specialized training and surgical expertise increases, HoLEP is expected to replace TURP and open surgery as the definitive treatment for large prostates.

**Figure 2:**
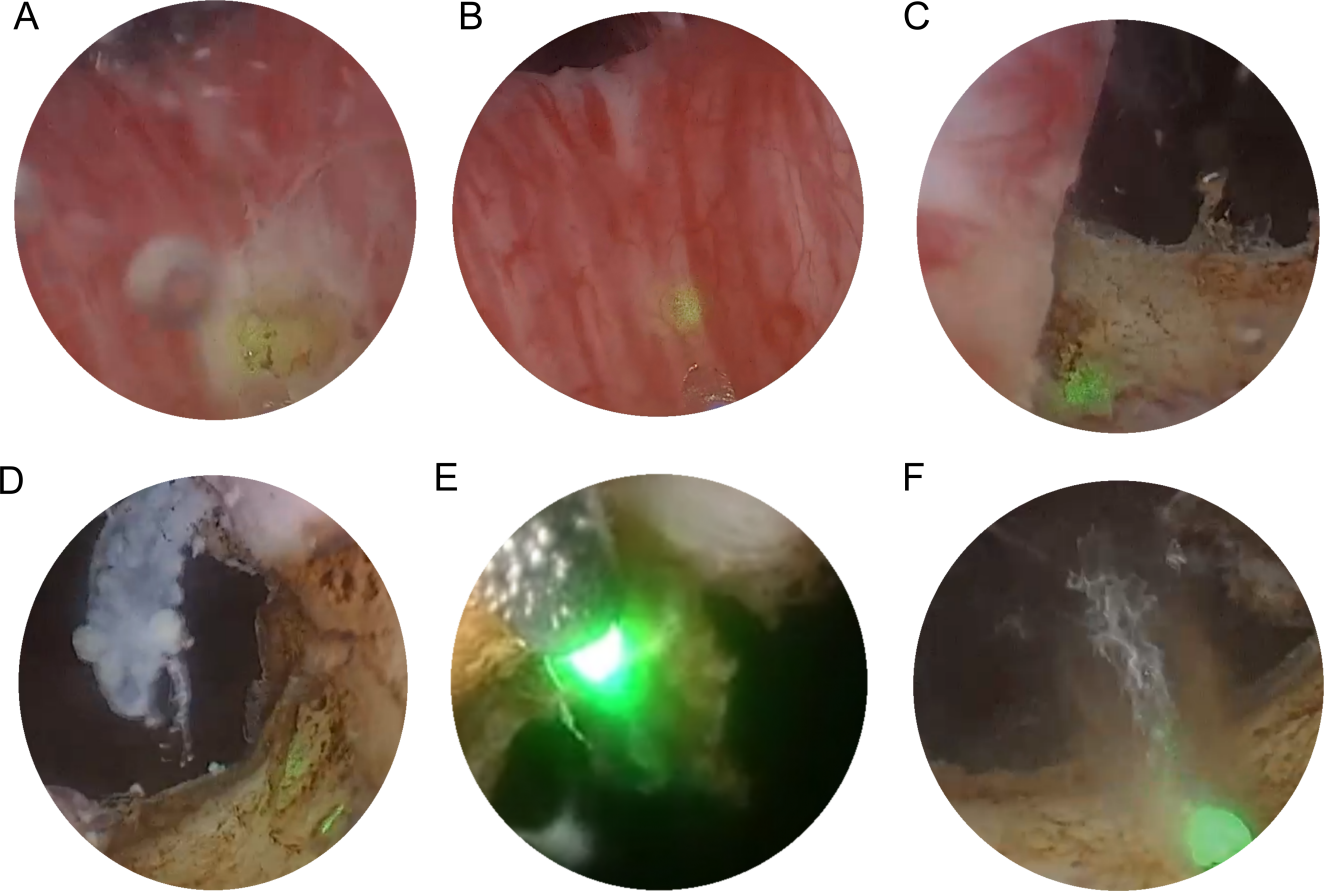
Transurethral holmium laser resection of prostate. **(A)** two deep incisions at the 5 o’clock and 7 o’clock positions at the bladder neck using the Holmium : YAG laser to define the enucleation plane. **(B)** If the median lobe is significantly enlarged, it is enucleated first. **(C, D)** After addressing the median lobe, attention is shifted to the lateral lobes. **(E, F)** The final phase of tissue removal focuses on the apex of the prostate, where the tissue near the verumontanum is carefully dissected.

HoLEP and TURP share similar surgical principles. The surgical procedure is shown in **Figure 2**. The surgeon begins the HoLEP procedure by making two deep incisions at the 5 o’clock and 7 o’clock positions at the bladder neck using the Holmium: YAG laser to define the enucleation plane. These trenches serve as anatomical landmarks, marking the boundary between the adenoma (enlarged prostate tissue) and the surgical capsule, allowing for precise enucleation. The laser is used in pulsed mode, ensuring controlled tissue cutting with minimal bleeding. If the median lobe is significantly enlarged, it is addressed first to relieve bladder outlet obstruction. The surgeon carefully dissects the median lobe from the capsule using laser energy, progressively separating it from surrounding structures. Once fully detached, the median lobe is pushed into the bladder, creating a wide, funnel-shaped opening to improve urinary flow. The procedure then continues with the enucleation of the lateral lobes, using a proximal-to-distal sweeping motion to achieve a controlled dissection. The laser precisely separates the lateral lobes from the capsular plane, ensuring complete detachment while preserving key anatomical structures. In the final phase, the apical tissue is carefully enucleated to ensure that no obstructive tissue remains. Special attention is given to the verumontanum to avoid damage to the external urinary sphincter, which is crucial for maintaining urinary continence. Once all lobes are fully enucleated, they are pushed into the bladder for further processing. A morcellator is then inserted through the resectoscope to fragment the enucleated prostate tissue into small pieces, which are subsequently suctioned out, completing the enucleation process. To ensure hemostasis and minimize the risk of postoperative bleeding, laser coagulation is applied to any bleeding sites. Finally, a three-way Foley catheter is inserted for continuous bladder irrigation, which is typically removed within 12 – 24 hours postoperatively (95).

Clinical studies consistently demonstrate that patients undergoing HoLEP experience a significant improvement in IPSS, a standardized measure of urinary symptom severity. Research shows that HoLEP leads to a reduction in IPSS scores by 70 - 80% within the first few months after surgery, with sustained improvement observed over long-term follow-ups. The primary mechanism behind this relief is the complete enucleation of the obstructive adenomatous prostate tissue, which effectively reduces bladder outlet obstruction and improves urinary flow dynamics. Compared to medical therapy, HoLEP provides more immediate and sustained relief from symptoms such as nocturia, urgency, hesitancy, weak urinary stream, and incomplete bladder emptying (96). One of the most significant advantages of HoLEP over other minimally invasive treatments is its effectiveness in reducing prostate volume, especially in patients with large prostates (>80–100 mL). HoLEP achieves near-total removal of the adenoma, leading to an average prostate volume reduction of 50 - 70% postoperatively (96). This extensive tissue removal translates to lower retreatment rates compared to other techniques, as residual adenoma is minimal. Additionally, the durability of HoLEP outcomes has been demonstrated in studies with 10 – 15 years of follow-up, where the risk of symptomatic recurrence remains very low. Unlike 5-ARIs used in medical management, which shrink the prostate gradually over several months, HoLEP provides immediate anatomical decompression, making it especially suitable for patients with severe urinary retention or bladder decompensation (97). HoLEP has been recognized for its excellent long-term durability, with studies showing low retreatment rates (<2% over 10 years). This is in contrast to TURP, where up to 10 - 15% of patients may require a secondary procedure within a decade due to residual or regrowth of adenomatous tissue. HoLEP’s ability to completely remove the obstructive adenoma at the surgical capsule level minimizes the risk of recurrence. Furthermore, rates of late complications, such as bladder neck contracture and urethral stricture, are comparable to or even lower than those seen with TURP. Patients who undergo HoLEP also report high satisfaction rates, as measured by validated QoL questionnaires (96).

### 3.3 Introduction of prostatic urethral lift procedure

The Prostatic Urethral Lift (PUL), commercially known as UroLift, is a minimally invasive procedure designed to relieve LUTS caused by BPH. Unlike traditional surgical interventions such as TURP or HoLEP, which involve the removal of prostate tissue, PUL preserves the prostate while mechanically widening the urethra. This is achieved by implanting permanent transprostatic devices, which pull the enlarged prostate lobes apart, creating a more open urethral passage to improve urinary flow. PUL is a breakthrough in BPH treatment as it offers immediate symptom relief with minimal downtime and fewer complications compared to traditional surgeries. This technique is particularly beneficial for men who: Seek a less invasive option than TURP or HoLEP. Want to preserve sexual function, as it does not impact ejaculation or erectile function. Have moderate prostate enlargement (30 – 80 mL) and are dissatisfied with medication but do not wish to undergo surgery (98–100). Are at higher surgical risk due to bleeding disorders or anticoagulant use.

**Figure 3:**
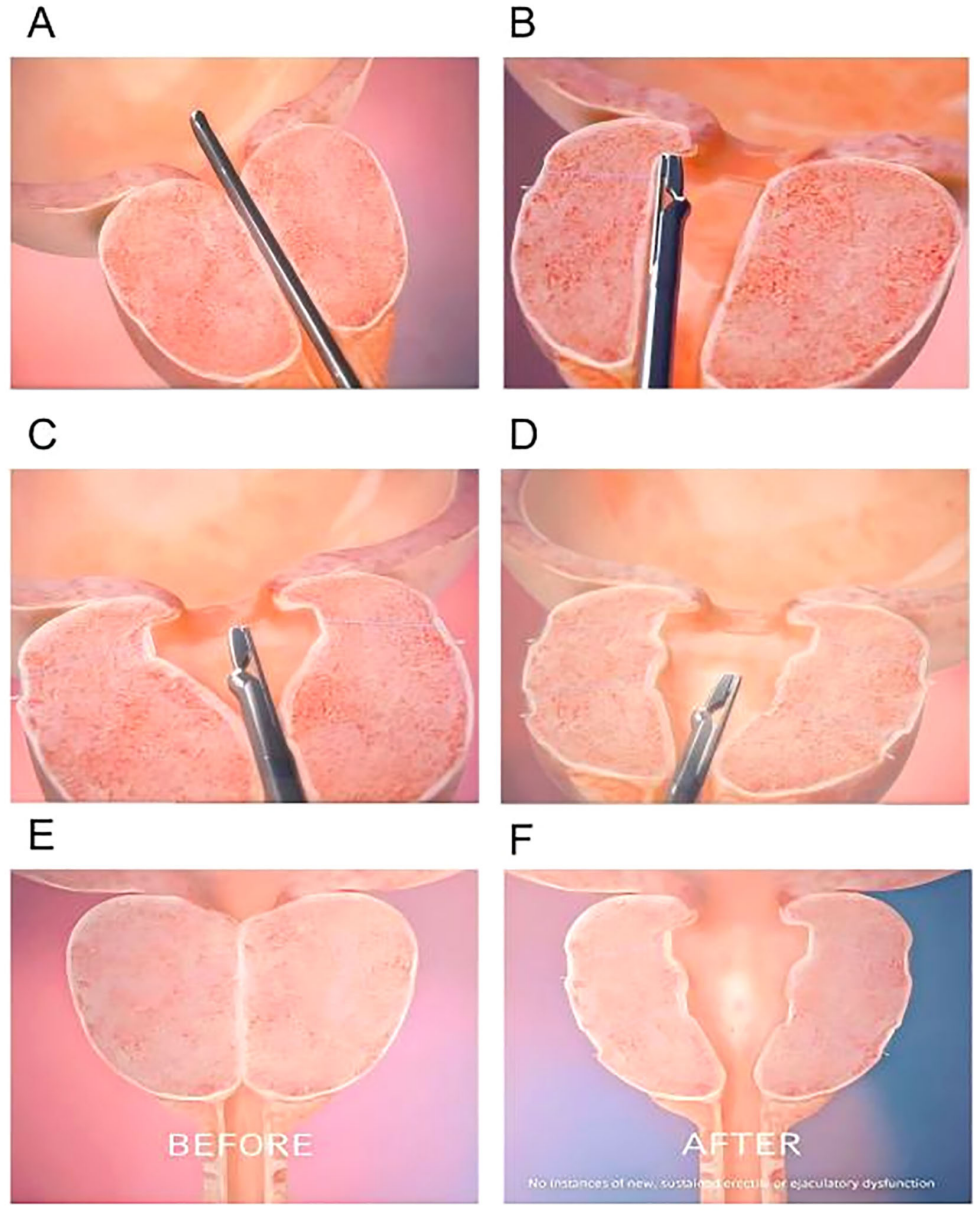
Step-by-step surgical technique for PUL. **(A)** A rigid cystoscope is inserted through the urethra to visualize the prostatic urethra and bladder neck. **(B)** Positions the implant delivery device against the prostatic urethra at the targeted location. Put a capsular tab to anchor the implant in the outer prostate tissue. **(C)** The second implant is placed on the opposite lateral lobe, mirroring the first placement. **(D)** A urethral end piece that secures the implant in the prostatic urethra. **(E, F)** Schematic of the prostate gland before and after implantation.

The PUL procedure begins with inserting a rigid cystoscope through the urethra to provide direct visualization of the prostatic urethra and bladder neck. The cystoscope allows the surgeon to assess the degree of prostatic obstruction and identify the optimal locations for implant placement. Once the prostatic lobes are evaluated, a dedicated implant delivery device is introduced via the working channel of the cystoscope to accurately position the implants. The UroLift implants, which are used in PUL, consist of three main components: a capsular tab that anchors the implant in the outer prostate tissue, a polyethylene terephthalate (PET) suture that provides tension to hold the lobes apart, and a urethral end piece that secures the implant in the prostatic urethra. Using the implant delivery device, one implant at a time is deployed into the lateral lobes of the prostate. These implants apply mechanical tension, pulling the obstructing prostate lobes apart and creating a wider urethral lumen, leading to improved urinary flow. The number of implants required depends on prostate size and degree of obstruction, with most patients receiving four to six implants(**Figure 3**). Unlike traditional resective procedures like TURP or HoLEP, which involve tissue removal, PUL mechanically repositions the prostate tissue without cutting, cauterizing, or vaporizing it. This results in immediate symptomatic relief, as the implants instantly create a widened prostatic urethral passage, without necrosis, scarring, or retraction of prostate tissue, thereby preserving the natural prostate anatomy. One of the major benefits of PUL is the preservation of sexual function, as it has no impact on ejaculation or erectile function, unlike TURP, which frequently results in retrograde ejaculation (101). Furthermore, unlike TURP and HoLEP, where post-procedural catheterization is standard, most PUL patients do not require prolonged catheter use. Since no heat or resection is involved, post-operative bleeding and urinary retention risks are minimal (102, 103). Some patients may require a short-term Foley catheter for a few hours, but most are catheter-free immediately after surgery. As a minimally invasive procedure, PUL typically takes 10 – 15 minutes and is performed on an outpatient basis under local anesthesia, sedation, or light general anesthesia. Most patients are discharged the same day and can resume normal activities within 24 – 48 hours. The absence of deep tissue trauma allows for faster recovery and a lower risk of complications compared to TURP and laser-based therapies (103).

### 3.4 Introduction of prostatic artery embolization

PAE, first described in 2000, is a minimally invasive, image-guided endovascular procedure that has emerged as an alternative to traditional surgical treatments for BPH. PAE is performed by interventional radiologists, who use catheter-based techniques to selectively embolize the prostatic arteries, reducing blood flow to the prostate and leading to its gradual shrinkage. This reduction in prostate volume ultimately alleviates LUTS associated with BPH (104).

**Figure 4:**
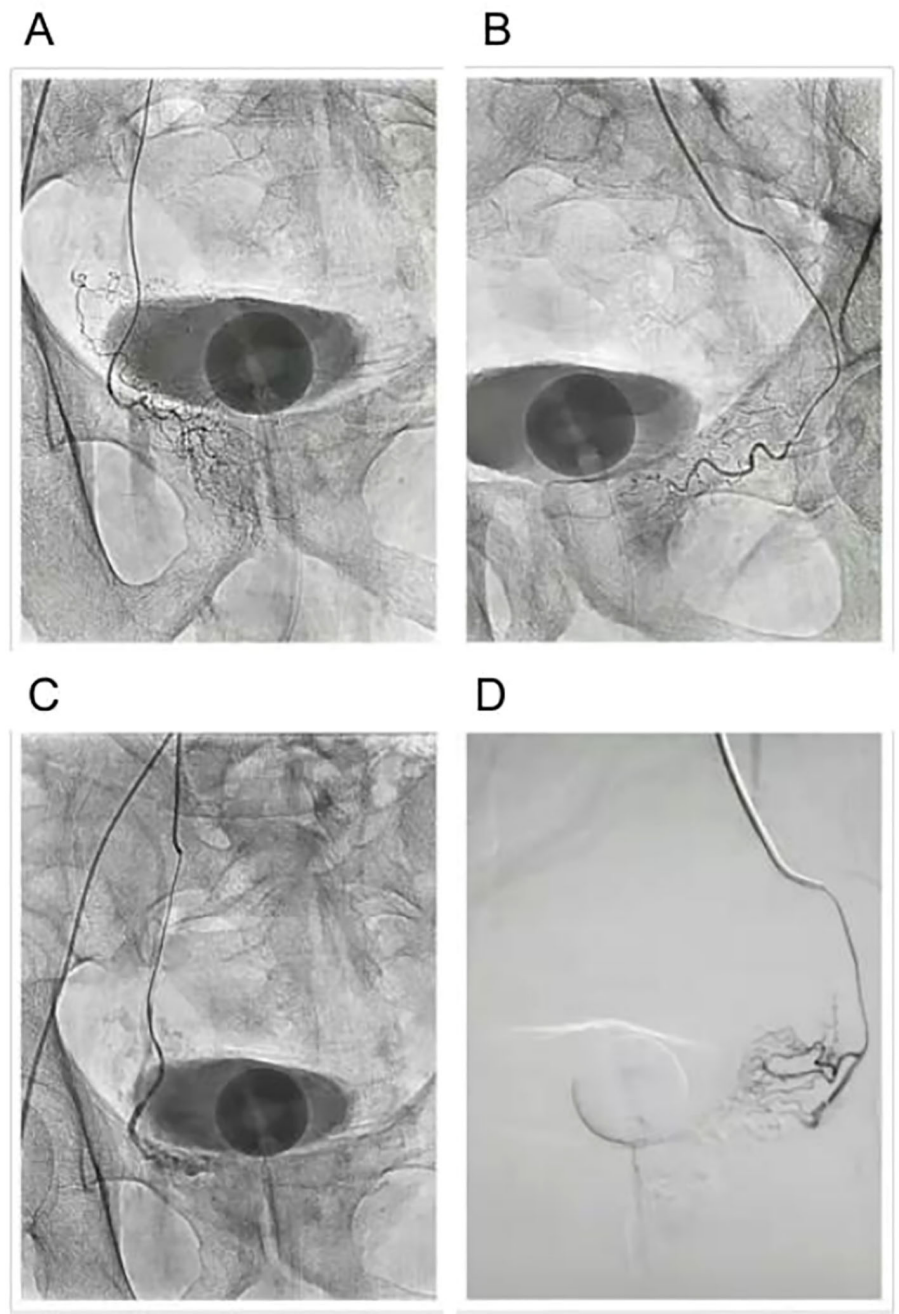
Prostatic arterial embolization (PAE) procedure. **(A)** Before embolization of the left prostatic artery. **(B)** Before embolization of the right prostatic artery. **(C)** After embolization of the left prostatic artery. **(D)** After embolization of the right prostatic artery.

The procedure begins with catheterization of the prostatic arteries, where a small catheter is inserted into the femoral or radial artery and advanced under fluoroscopic guidance to reach the prostatic arterial supply. Once the catheter is correctly positioned, embolization using microparticles follows, where embolic agents such as polyvinyl alcohol (PVA) particles or microspheres are injected into the prostatic arteries. These particles travel through the arterial system and block blood flow to the prostate tissue, effectively reducing the oxygen and nutrient supply needed for prostate growth. Over the course of weeks to months, the ischemic prostate tissue undergoes necrosis, leading to a gradual reduction in prostate volume and subsequent relief of urinary symptoms (**Figure 4**) (105).

Because PAE does not involve resecting, cutting, or vaporizing prostate tissue, it presents several advantages over conventional surgical techniques such as TURP or Holmium Laser Enucleation of the Prostate HoLEP. One of its most significant benefits is a lower risk of complications, particularly sexual dysfunction, incontinence, and retrograde ejaculation, which are commonly associated with more invasive BPH treatments. Additionally, PAE is performed under local anesthesia, making it a viable option for elderly patients or those with multiple comorbidities who may not be ideal candidates for general anesthesia or major surgery. Due to its minimally invasive nature, patients undergoing PAE typically experience a shorter recovery time, fewer hospitalizations, and a reduced risk of post-procedure bleeding compared to TURP or HoLEP (106). While PAE provides a gradual improvement in symptoms, rather than the immediate relief offered by TURP, its long-term durability and safety profile make it an increasingly popular choice for men seeking a less invasive and lower-risk alternative to traditional prostate surgery (107).

### 3.5 Introduction of prostatic steam ablation

Prostatic Steam Ablation, commonly referred to as water vapor thermal therapy, is a minimally invasive treatment designed to alleviate LUTS caused by BPH. One of the most well-known forms of this procedure is Rezūm therapy, which utilizes radiofrequency-generated water vapor to ablate hyperplastic prostate tissue (108). This therapy represents a paradigm shift in BPH treatment, offering an alternative to medications and traditional surgical interventions. Unlike TURP or HoLEP, which involve mechanical tissue removal, steam vaporization delivers controlled thermal energy into the prostate. The vapor induces cellular destruction, leading to a gradual reduction in prostate volume over the following weeks. This process helps relieve bladder outlet obstruction while preserving sexual function and minimizing perioperative risks (109).

**Figure 5:**
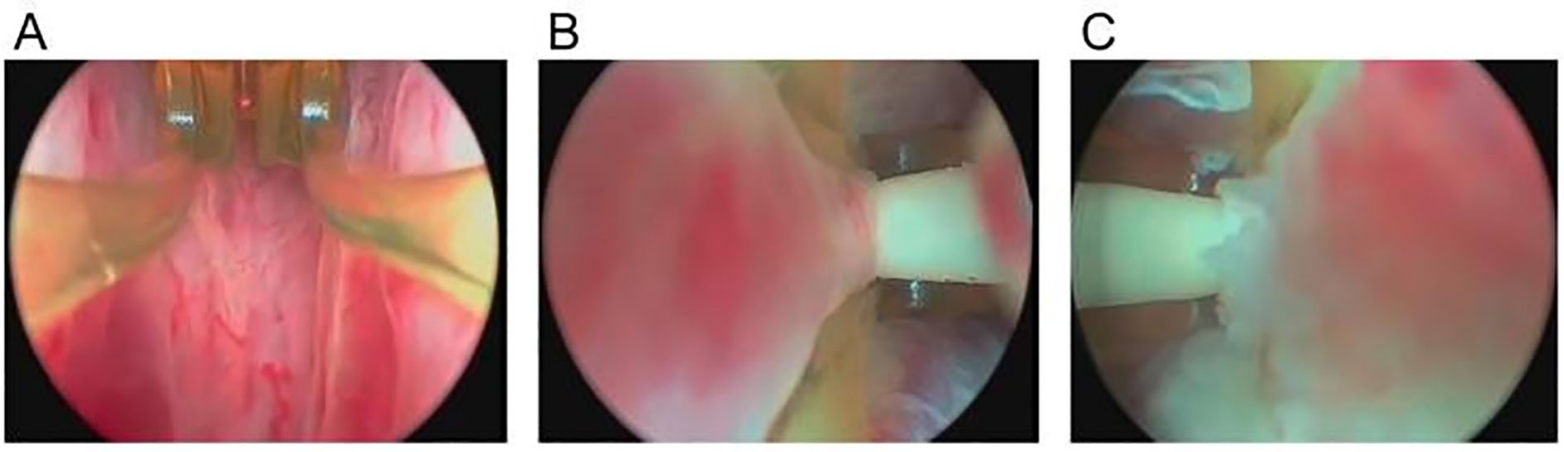
Endoscopic views of prostate steam ablation (Rezūm) procedure. **(A)** The electrode is inserted into the prostate tissue. The bilateral structures are symmetrical, and the vascular network of the prostate mucosa is visible. **(B)** The prostate tissue appears congested as steam is delivered through the specialized catheter into the left hyperplastic tissue, leading to localized tissue denaturation. **(C)** The steam is released through a specialized catheter into the hyperplastic prostate tissue on the right side, causing localized tissue denaturation.

The procedure begins with a transurethral delivery system, where a small handheld device is inserted through the urethra. A radiofrequency-powered generator heats water, converting it into steam, which is then injected directly into the obstructive prostate tissue via a needle deployment system. This minimally invasive therapy is typically performed under local anesthesia in an outpatient setting, making it a convenient alternative to traditional surgeries. Once injected into the prostate, sterile water vapor rapidly condenses upon contact with the cooler prostate tissue, releasing thermal energy at approximately 103 °C. This heat denatures proteins within the prostate cells, leading to immediate coagulative necrosis. Over the next several weeks, the body’s immune response and inflammatory mechanisms work to clear the necrotic tissue. As the damaged prostate tissue is gradually reabsorbed, the prostate volume decreases, reducing bladder outlet obstruction and improving urinary flow. The urethral lumen progressively widens as necrotic tissue is absorbed, alleviating symptoms over time. Unlike traditional surgical approaches such as TURP, which immediately removes prostate tissue, steam therapy allows for a gradual remodeling process. Patients typically begin to experience symptom relief within a few weeks post-procedure. Additionally, this technique preserves key anatomical structures, including the bladder neck and external urinary sphincter, significantly reducing the risk of complications such as retrograde ejaculation and urinary incontinence, which are commonly associated with TURP (**Figure 5**).

After Rezūm procedure, proper post-procedural management is essential for recovery. Most patients require a Foley catheter for 2 to 7 days to assist bladder drainage, with removal during a follow-up visit. Mild discomfort, urinary urgency, and hematuria are common in the first week and can be managed with hydration, NSAIDs, alpha-blockers, and antibiotics if necessary. Urinary symptoms may temporarily worsen but generally improve within 2 – 6 weeks, with maximum benefits seen by 3 months. Patients should avoid heavy lifting, vigorous exercise, and sexual activity for 2 – 4 weeks. Follow-up appointments at 2 weeks, 3 months, and 6 months help monitor progress. The procedure preserves sexual function and has a lower risk of incontinence compared to surgical interventions (110, 111).

After a prostate steam ablation (Rezūm) procedure, proper post-procedural management is essential for recovery. Most patients require a Foley catheter for 2 to 7 days to assist bladder drainage, with removal during a follow-up visit (112). Mild discomfort, urinary urgency, and hematuria are common in the first week and can be managed with hydration, NSAIDs, alpha-blockers, and antibiotics if necessary. Urinary symptoms may temporarily worsen but generally improve within 2 – 6 weeks, with maximum benefits seen by 3 months. Patients should avoid heavy lifting, vigorous exercise, and sexual activity for 2 – 4 weeks (112). Follow-up appointments at 2 weeks, 3 months, and 6 months help monitor progress. The procedure preserves sexual function and has a lower risk of incontinence compared to surgical interventions (111).

### 3.6 Introduction of high-energy water jet ablation

High-energy water jet ablation, commonly known as Aquablation, is an advanced robotically controlled, minimally invasive surgical therapy (MIST) for treating BPH. It uses a high-velocity, high-pressure water jet to precisely remove obstructive prostate tissue while preserving surrounding structures and minimizing complications such as sexual dysfunction and urinary incontinence. The Aquablation procedure is performed using the AquaBeam^®^ Robotic System, an advanced robotic-assisted platform designed to deliver precise, heat-free resection of the prostate in men with BPH. This system integrates three key technological components—real-time ultrasound imaging, automated robotic control, and high-energy water jet technology—to ensure maximal efficacy while minimizing complications (113). Unlike traditional BPH treatments that rely solely on endoscopic visualization, the AquaBeam^®^ system integrates real-time ultrasound guidance, providing a three-dimensional (3D) live view of the prostate (114). This imaging enables precise mapping of the prostatic tissue, allowing the surgeon to customize the treatment zone while avoiding key anatomical structures such as the bladder neck, external sphincter, and ejaculatory ducts. This personalized treatment planning significantly reduces the risks of incontinence, retrograde ejaculation, and excessive tissue removal, ensuring a better functional outcome (115). Additionally, the AquaBeam^®^ system utilizes automated robotic technology, eliminating manual variability seen in traditional surgical procedures such as TURP and HoLEP.

**Figure 6:**
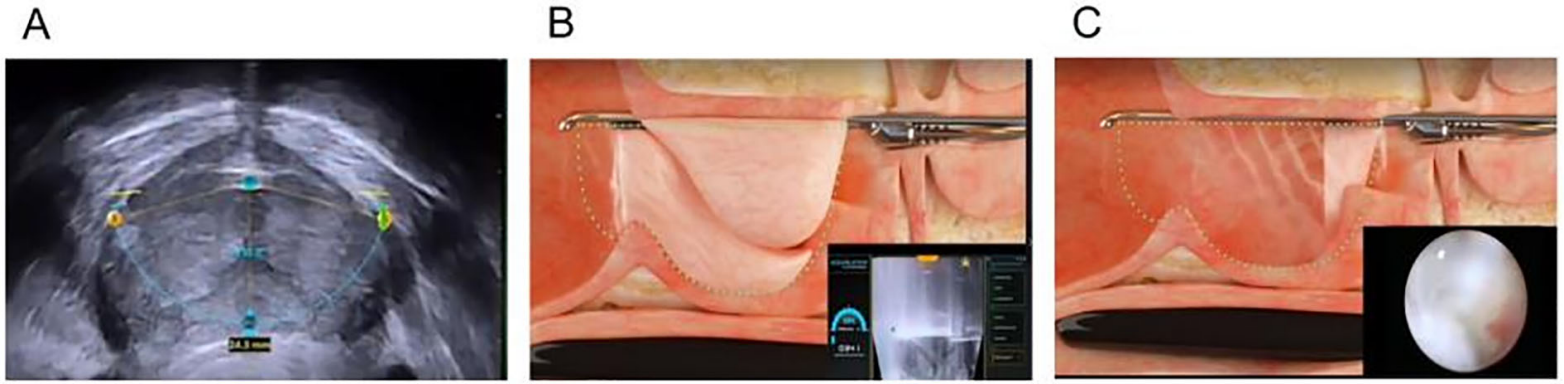
Aquablation for BPH. **(A)** Preoperatively, color Doppler ultrasound is used to locate the prostate and assess the extent of tissue that needs to be resected. **(B)** The water jet ablates the predetermined prostate tissue within the planned resection area. **(C)** The water jet ablation of the prostate.

The procedure follows a step-by-step approach for optimal outcomes. First, during the planning and customization phase, the surgeon utilizes live ultrasound imaging to map the treatment area, selecting the prostate tissue to remove while carefully avoiding key structures such as the bladder neck and external sphincter. Next, during the water jet ablation phase, the high-energy water jet is delivered robotically to the predefined area, removing hyperplastic prostate tissue in just a few minutes. Because the energy is non-thermal, it significantly reduces the risk of heat-related complications, such as fibrosis and nerve damage. Finally, in the hemostasis and recovery phase, unlike traditional procedures like TURP or HoLEP, which rely on electrocautery for bleeding control, Aquablation may require additional hemostatic techniques, such as balloon tamponade or electrocautery to manage bleeding (**Figure 6**). A catheter is typically placed for 24 – 48 hours post-procedure to reduce swelling and manage mild bleeding. This approach ensures a minimally invasive yet highly effective treatment for BPH with faster recovery and reduced complications compared to traditional surgical methods.

Aquablation offers several key advantages over traditional BPH treatments, making it a highly effective and minimally invasive option. Unlike TURP, HoLEP, or GreenLight laser, which rely on electrocautery or laser energy, Aquablation avoids thermal damage, thereby reducing the risk of nerve damage (which is crucial for erectile function) and excessive fibrosis and scarring, which can lead to stricture formation (116). The robotic-controlled system, combined with real-time ultrasound imaging, allows for precise, personalized treatment planning, ensuring improved accuracy and reproducibility across different patients. One of its most significant advantages is the preservation of ejaculatory function, as many BPH treatments, particularly TURP and HoLEP, result in retrograde ejaculation in 50 – 80% of patients, whereas Aquablation presents a lower risk of ejaculatory dysfunction, making it an ideal choice for sexually active men (117). Additionally, Aquablation is highly effective for large prostates, as TURP is typically limited to prostates under 80g, whereas Aquablation can successfully treat prostates over 100g, providing a viable alternative to open simple prostatectomy (118). Furthermore, the procedure is significantly faster, with the actual water jet ablation taking only 5 minutes, and most patients stay just one night in the hospital, experiencing faster recovery compared to TURP or HoLEP (119). These benefits make Aquablation a groundbreaking advancement in the minimally invasive treatment of BPH.

### 3.7 Introduction to prostatic stents

A prostatic stent is a small, cylindrical, tube-like medical device that is inserted into the prostatic urethra to maintain its patency (openness) and facilitate unobstructed urinary flow. It is commonly used in men with BPH or other LUTS where traditional treatments, such as medications or surgical interventions, may not be suitable (120). The prostatic stent functions through mechanical expansion, bypassing blockage from an enlarged prostate to restore urinary flow. Once placed in the prostatic urethra, it expands and applies outward pressure on surrounding tissue, widening the urethral lumen and improving bladder emptying (121).This relieves symptoms such as urinary retention, urgency, and weak stream. Temporary stents remain for weeks to months, while permanent stents integrate into the urethra for long-term patency. Most patients experience immediate relief, making stents a minimally invasive option for managing BPH and lower urinary tract obstruction (122).

Prostatic stents are made from three main material types, each with distinct properties. Metallic stents (e.g., Nitinol, stainless steel, gold-plated alloys) offer high durability and long-term patency, suitable for permanent use. Polymer-based stents (e.g., silicone, polyurethane, PLA) provide flexibility and biocompatibility with less irritation but lower durability. Biodegradable stents (e.g., PLGA, magnesium alloys) dissolve over time, avoiding removal and reducing long-term complications. Material choice depends on patient needs, obstruction severity, and intended duration (**Table 7**) (120, 122).

**Table 7:** Common materials, advantages and disadvantages of prostate stents.

Material	Type	Advantages	Disadvantages	Examples
Nitinol (Nickel-Titanium Alloy) (121–124)	Metallic (Permanent)	Superelasticity & Shape Memory – Expands and conforms well to the urethral shape.	Risk of migration – If not properly anchored.	Memokath™ 028 Prostatic Stent
		Corrosion-resistant – High durability in body fluids.	May require removal due to long-term irritation.	
		Minimal encrustation risk – Compared to stainless steel.	Nickel sensitivity/allergy risk in some patients.	
		Biocompatible – Well-tolerated in most patients.		
Stainless Steel (121–124)	Metallic (Permanent)	Strong and durable – Provides long-term urethral patency.	Prone to encrustation – Higher risk of stone formation.	UroLume™ Stent
		Lower cost than nitinol.	Rigid – Can cause discomfort or tissue damage.	
		Effective in patients with severe BPH obstruction.	Difficult to remove if complications arise.	
Gold-Coated Stents (121–124)	Metallic (Permanent)	Higher biocompatibility – Gold reduces inflammatory response.	Expensive – Gold increases material cost.	Gianturco-Rosch Metallic Stent
		Lower encrustation risk – Compared to stainless steel.	Risk of migration – If not properly positioned.	
		Radiopaque – Easily visible in imaging for positioning.		
Silicone (121–124)	Polymer (Temporary & Permanent)	Flexible & soft – Reduces discomfort.	Lower mechanical strength – May collapse under pressure.	Spanner™ Temporary Prostatic Stent
		Minimizes tissue trauma – Non-reactive material.	Less effective in highly obstructed prostates.	
		Lower encrustation risk than metallic stents.		
Polyurethane (PU) (121–124)	Polymer (Temporary & Permanent)	Elastic & durable – Withstands compression.	Higher risk of bacterial colonization – Can lead to infections.	Optilume™ Drug-Coated Balloon Stent
		Good biocompatibility – Reduces irritation.	Potential for biofilm formation – Requires careful monitoring.	
		Customizable for different stent shapes and sizes.		
PLA (121–124)	Biodegradable (Temporary)	Dissolves over time – No need for removal.	Lower mechanical strength – Might degrade too quickly.	VESAir™ Biodegradable Stent
		Minimizes long-term complications.	Not suitable for severe obstructions.	
		Biocompatible and reduces inflammation.	Limited clinical data on long-term effectiveness.	
PLGA (Polylactic-Co-Glycolic Acid) (120–125)	Biodegradable (Temporary)	Controlled degradation rate – Tailored for short-term use.	Potential for incomplete degradation – May leave debris.	Artemis™ Bioabsorbable Stent
		No need for removal – Reduces follow-up interventions.	Limited availability – Still under clinical research.	
		Reduces risk of stent-related infections.		
Magnesium-based Stents (120)	Biodegradable (Temporary)	Slowly dissolves over time – Avoids long-term complications.	Still under investigation – Limited clinical studies.	MAGiC™ Resorbable Stent (Experimental)
		Promotes natural healing of the urethra.	May degrade unpredictably – Stability concerns.	
		Lower inflammation risk – Compared to synthetic polymers.		

**Figure 7:**
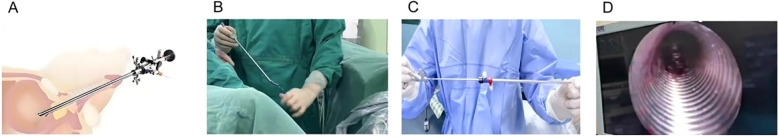
Prostatic stent placement procedure. **(A)** A schematic illustration of the prostatic stent placement procedure. **(B)** Under sterile conditions, the doctor dilates the urethra. **(C)** The stent is preloaded onto the system and is positioned before insertion. **(D)** The stent maintains urethral patency by mechanically expanding and compressing the hyperplastic prostate tissue.

Prostatic stent insertion process. Patient Preparation, The patient is positioned in the lithotomy position (legs elevated) on the surgical table. Local anesthesia or mild sedation is administered to minimize discomfort. A cystoscope (a thin, flexible or rigid tube with a camera and light) is inserted into the urethra through the penis to visualize the prostatic urethra and bladder. Stent Selection and Positioning, the urologist selects an appropriate stent type (temporary or permanent) based on the severity of BPH and the patient’s overall condition. Using endoscopic guidance, the stent delivery system is inserted into the prostatic urethra. The target location is identified, ensuring the stent is positioned just above the external urinary sphincter to avoid complications. Deployment of the Stent, the stent is slowly expanded inside the urethra using self-expanding mechanisms (e.g., nitinol-based stents expand due to 55°C temperature). Some stents require manual expansion using a balloon catheter to push the prostate tissue outward. The stent secures itself in place by conforming to the prostatic urethra’s anatomy, ensuring stability (**Figure 7**).

Although several randomized controlled trials (RCTs) have evaluated prostatic stents for benign prostatic hyperplasia (BPH) and lower urinary tract obstruction (LUTO), the current body of evidence is limited in both scale and methodological quality. In a systematic review of 27 studies, only 11.1% were RCTs, most being single-center (81.5%) with small sample sizes (median 42 patients) and short follow-up (median 12 months) (120). Existing RCTs, such as the multicenter MT - 02 trial and device-specific studies on the Spanner stent, consistently demonstrate short-term benefits—for example, catheter-free rates of ~83% at three months, IPSS reductions of 9 – 10 points, and Qmax gains of ~6 mL/s—but lack long-term (>12 months) durability data and head-to-head comparisons with gold-standard surgical options like transurethral resection of the prostate (TURP) or laser procedures. However, the treatment failure rate—stent removal or repositioning—was 14.8% at a median 12-month follow-up (123). A large single-center series of 150 intraprostatic spiral stents also reported >20% symptom recurrence at 12 months due to migration, encrustation, and tissue ingrowth (124). The complication burden is substantial: urinary tract infection (17.2%), calcification (12.6%), irritative symptoms (12.2%), and acute urinary retention (10.4%) were common, with older permanent stents like UroLume showing migration rates of 10 – 36% and encrustation up to 30% (125).

In summary, while available RCTs and observational studies indicate that prostatic stents can achieve short-term symptom relief and catheter independence in carefully selected, high-risk patients, the lack of large-scale, long-term, high-quality RCTs and the persistence of significant device-related complications limit their endorsement as a routine alternative to established surgical treatments for BPH/LUTO.

### 3.8 Summary of minimally MISTs for BPH and future directions

MISTs have revolutionized BPH treatment, offering effective symptom relief with reduced morbidity and faster recovery compared to traditional open prostatectomy. TURP and HoLEP remain the gold standards for moderate-to-severe BPH, particularly for larger prostates, while newer techniques such as Aquablation and Rezūm provide less invasive alternatives with fewer sexual side effects. PUL and prostatic stents are ideal for preserving ejaculation, whereas PAE serves as an attractive non-surgical option for high-risk patients. The future of BPH treatment is being shaped by robotic-assisted and AI-driven procedures, enhancing surgical precision, tissue-sparing techniques, and real-time intraoperative decision-making. Hybrid therapies that combine TURP with laser technologies (e.g., GreenLight, HoLEP) or PAE with thermal therapies may offer superior long-term symptom relief with lower complication rates (**Table 8**). Meanwhile, the development of non-thermal energy-based treatments, such as pulsed electromagnetic fields (PEMFs) or radiofrequency modulation, presents promising non-destructive approaches to prostate volume reduction. Biodegradable drug-eluting stents and smart implantable devices could further optimize temporary relief while reducing migration risks. Additionally, AI-driven patient selection algorithms will play a pivotal role in customizing treatment plans, analyzing prostate volume, symptom severity, and patient comorbidities to select the most suitable intervention. Ejaculatory function preservation techniques, such as Ejaculatory Hood-Sparing TURP and refined Aquablation strategies, continue to evolve, addressing concerns related to sexual dysfunction. As robotics, regenerative medicine, and machine learning advance, the landscape of BPH treatment will become safer, more effective, and more tailored to individual patient needs, ensuring optimal outcomes with minimal side effects.

**Table 8:** Minimally invasive treatments for BPH: comparative overview.

Treatment	Procedure Type	Anesthesia	Invasiveness	Indications	Contraindications	Advantages	Disadvantages	Prostate Size Suitability	Prostate Volume Reduction	Symptom Relief	Hospital Stay	Risk of Bleeding	Sexual Function Impact	Risk of Incontinence	Catheterization Needed	Durability of Treatment	LUTS Improvement Rate	IPSS Score Improvement	QoL Score Improvement
TURP (92, 101)	Endoscopic Resection	Spinal/General	Moderate	Moderate-to-severe LUTS, failed medication therapy, urinary retention	Large prostate (>100g), severe bleeding disorders	Gold standard, effective, long-term symptom relief	Risk of bleeding, retrograde ejaculation, hospital stay	<80g (Best for 30-80g)	High (40-60%)	Significant	1-3 days	High	Moderate (50-75% retrograde ejaculation)	Moderate (2-6%)	Yes (2-5 days)	Long-term (10+ years)	80-90%	14-18 points	2-3 points
Holep (91, 95, 101, 103)	Laser-Based	Spinal/General	Moderate	Large prostates (>50g), recurrent urinary retention, previous failed TURP	Patients unable to tolerate anesthesia, small prostate (<30g)	Suitable for large prostates, less bleeding than TURP	Requires specialized equipment, longer learning curve	<100g (Best for 40-100g)	High (50-70%)	Significant	1-2 days	Moderate	Moderate (30-60% retrograde ejaculation)	Low (1-3%)	Yes (1-3 days)	Long-term (10+ years)	80-90%	14-18 points	2-3 points
PUL (Prostatic Urethral Lift, UroLift**®**) (98, 109)	Implant-Based	Local/Sedation	Low	Mild-to-moderate LUTS, patients desiring ejaculation preservation	Large prostates (>80g), significant obstruction	Minimally invasive, preserves sexual function	Less effective in large prostates, potential device migration	<80g (Best for 30-70g)	No tissue removal	Moderate	Same day	Low	Low (Minimal effect on ejaculation)	Very low (<1%)	No or <24 hours	Moderate (5-7 years)	60-70%	8-12 points	1-2 points
PAE (105–107)	Endovascular	Local	Low	High-risk patients, large prostates, anticoagulated patients	Severe LUTS, extensive arterial disease	Outpatient, no surgery, suitable for large prostates	Gradual symptom improvement, not covered by all insurance	>50g (Best for 60-120g)	Moderate (20-40%)	Gradual	Outpatient/Same day	Low	Low	Very low (<1%)	No	Moderate (5-8 years)	50-70%	8-14 points	1-2 points
Prostatic Artery Steam Ablation (Rezūm™) (110–112)	Steam-Based	Local/Sedation	Low	Moderate BPH, men desiring minimally invasive option	Severe obstruction, high prostate volume (>80g)	Minimally invasive, preserves ejaculation	Temporary symptom worsening, catheterization required	<80g (Best for 30-80g)	Moderate (20-40%)	Gradual	Same day	Low	Low	Low (<3%)	Yes (3-7 days)	Moderate (5+ years)	50-75%	8-15 points	1-2 points
High-Energy Water Jet Ablation (Aquablation**®**) (98, 112, 119)	Robotic Water Jet	Spinal/General	Moderate	Large prostates (>80g), men desiring ejaculation preservation	Severe bleeding disorders, previous urethral surgery	Preserves ejaculation, effective for large prostates	Requires robotic system, risk of temporary hematuria	30-150g	High (40-60%)	Significant	1-2 days	Moderate	Low (Preserves ejaculation)	Low (<2%)	Yes (1-2 days)	Long-term (10+ years)	70-85%	12-18 points	2-3 points
Prostatic Stents (121–123)	Stent-Based	Local	Very Low	High-risk patients unable to undergo surgery	Active UTI, high risk of stent migration	Quick, no anesthesia required, immediate symptom relief	Stent migration, encrustation, may need replacement	<100g (Best for high-risk patients)	No tissue removal	Immediate	Same day	Low	Low	Moderate (Migration risk)	No	Temporary or Long-Term	50-70%	6-12 points	1-2 points

## 4 Conclusion

BPH treatment has significantly evolved, transitioning from highly invasive surgical procedures to a combination of pharmacological therapy and MISTs that offer effective symptom relief with fewer complications and faster recovery. Medical therapy, including α-blockers, 5-ARIs, PDE5i, and combination regimens, remains the first-line treatment for mild-to-moderate BPH, providing symptom relief while delaying or preventing the need for surgical intervention. However, for patients with more severe symptoms or those who do not respond well to medications, MISTs such as Rezūm, Aquablation, UroLift, and PAE offer less invasive alternatives with a focus on reducing morbidity and preserving sexual function, while TURP and HoLEP remain the gold standard for larger prostates due to their high efficacy and long-term durability. The future of BPH management will be driven by robotic-assisted precision surgery, AI-guided treatment selection, hybrid therapies, and non-thermal, energy-based techniques aimed at minimizing side effects while optimizing clinical outcomes. Additionally, biodegradable stents, gene therapy, and regenerative medicine may further revolutionize prostate care by offering longer-lasting relief while reducing the need for repeat interventions. AI-driven predictive modeling will enhance patient-specific treatment planning, ensuring individualized approaches tailored to prostate size, symptom severity, and comorbidities. With these continuous advancements, BPH treatment is becoming increasingly personalized, safer, and more effective, integrating pharmacological, minimally invasive, and surgical options to provide optimal symptom control and preserve patient quality of life.
